# Latent-period stool proteomic assay of multiple sclerosis model indicates protective capacity of host-expressed protease inhibitors

**DOI:** 10.1038/s41598-019-48495-5

**Published:** 2019-08-28

**Authors:** Carlos G. Gonzalez, Stephanie K. Tankou, Laura M. Cox, Ellen P. Casavant, Howard L. Weiner, Joshua E. Elias

**Affiliations:** 1 Chemical and Systems Biology Department, Stanford University School of Medicine, Stanford, USA; 2 Ann Romney Center for Neurological Diseases, Brigham and Women’s Hospital, Harvard School of Medicine, Boston, MA USA; 3 Department Of Neurology, Icahn School Of Medicine at Mount Sinai, New York, NY USA; 4 Precision Immunology Institute, Icahn School Of Medicine at Mount Sinai, New York, NY USA; 5 Friedman Brain Institute, Icahn School Of Medicine at Mount Sinai, New York, NY USA; 6 Chan Zuckerberg Biohub, San Francisco California, USA

**Keywords:** Microbiome, Autoimmunity, Mass spectrometry, Predictive markers

## Abstract

Diseases are often diagnosed once overt symptoms arise, ignoring the prior latent period when effective prevention may be possible. Experimental autoimmune encephalomyelitis (EAE), a model for multiple sclerosis, exhibits such disease latency, but the molecular processes underlying this asymptomatic period remain poorly characterized. Gut microbes also influence EAE severity, yet their impact on the latent period remains unknown. Here, we show the latent period between immunization and EAE’s overt symptom onset is characterized by distinct host responses as measured by stool proteomics. In particular, we found a transient increase in protease inhibitors that inversely correlated with disease severity. Vancomycin administration attenuated both EAE symptoms and protease inhibitor induction potentially by decreasing immune system reactivity, supporting a subset of the microbiota’s role in modulating the host’s latent period response. These results strengthen previous evidence of proteases and their inhibitors in EAE and highlight the utility stool-omics for revealing complex, dynamic biology.

## Introduction

Both chronic and acute diseases are classically characterized by overt symptoms and their related molecular signatures. However, if these symptoms manifest gradually over time, diagnoses could be delayed and the window for effective therapeutic treatment may be missed. Thus, the latent period – the time period prior to overt symptom onset – represents an important period of disease progression. Indeed, focused characterization of a disease’s latent period can reveal biomarkers foretelling subsequent disease trajectory and severity, as well as highlight pathways for therapeutic targeting^1–3^.

However, the latent period’s biological significance and therapeutic potential is often hampered by an incomplete understanding of temporally significant events within this time frame. For instance, the peripheral limb weakness, decreased mental acuity, reduced bowel control, and other overt symptoms which characterize relapsing-remitting multiple sclerosis (MS) flares are the consequences of autoimmune activity against myelin proteins in the central nervous system. However, events that promote the disease’s asymptomatic periods or trigger eventual relapses remain poorly understood. A commonly used mouse model of MS, experimental autoimmune encephalomyelitis (EAE), also exhibits a latent period prior to disease manifestation. In the case of the mouse (C57BL/6J) EAE model, this period is characteristically 10–12 days, thus enabling focused study of this period.

While EAE’s latent period timeframe is well-known, the biological underpinnings of how it sets up the classical disease state are far less understood. One factor that has come to be associated with MS and EAE in the past decade is the gut microbiome. Indeed, previous research has shown MS patients harbor altered gut microbial communities compared to healthy controls^4–6^. In line with this evidence, EAE induction can be heavily influenced by the presence of specific microbial community members, although the host-microbiome interactions that lead to these results are not entirely delineated^7^.

Due to the gut’s proximity to microbes and its high degree of innate and adaptive immune activity, dynamically monitoring gut physiological activity prior to the manifestation of EAE stands to provide critical insights into EAE’s latent period. Stool is particularly useful due to its non-invasive collection and compatibility with large, longitudinal multi-omic studies that leverage the presence of host and microbe proteins, intact microbes, and metabolites^8^. Furthermore, because many proteins have well-characterized functional roles in host immunity, measuring systems-level proteomic changes in latent period EAE makes stool ideal for bridging our understanding of how microbes and the host immunity interact during EAE.

We designed the present study, the first report to focus on latent EAE using proteomics, to characterize changes in gut microbiota and extracellular gut proteomes during this understudied time period. We leveraged temporal 16S rRNA microbial sequencing and shotgun proteomics of mouse stool in order to measure the intricate interplay of host response, microbes, and disease. We found that microbes and extracellular proteins measured from stool were able to distinguish pre-symptomatic phases of EAE from non-diseased states to varying extents. Latent period 16S rRNA microbiome measurements did not reveal large-scale consistent patterns over time, however several genera exhibited modest changes in abundance. Shotgun proteomics revealed at least two unique latent period signatures associated with immune system suppression and subsequent inflammation. Among the protein classes driving these signatures were host-expressed protease inhibitors, which exhibited a pronounced post-immunization increase. Surprisingly, protease inhibitor levels inversely corresponded with eventual EAE symptom severity. Additionally, this work pointed to correlations between specific protease inhibitors and microbes prior to the onset of disease, providing a basis for further mechanistic studies focused on the interplay between immune regulation and microbiome composition. Lastly, we found that depleting a subset of microbes with the antibiotic vancomycin prior to immunization broadly attenuated the increased protease inhibitor levels, suggesting microbes sensitive to this antibiotic were responsible for immune system priming prior to immunization.

## Results

### Latent period microbe community structures do not obviously reflect immunization or EAE symptoms

**Figure 1:**
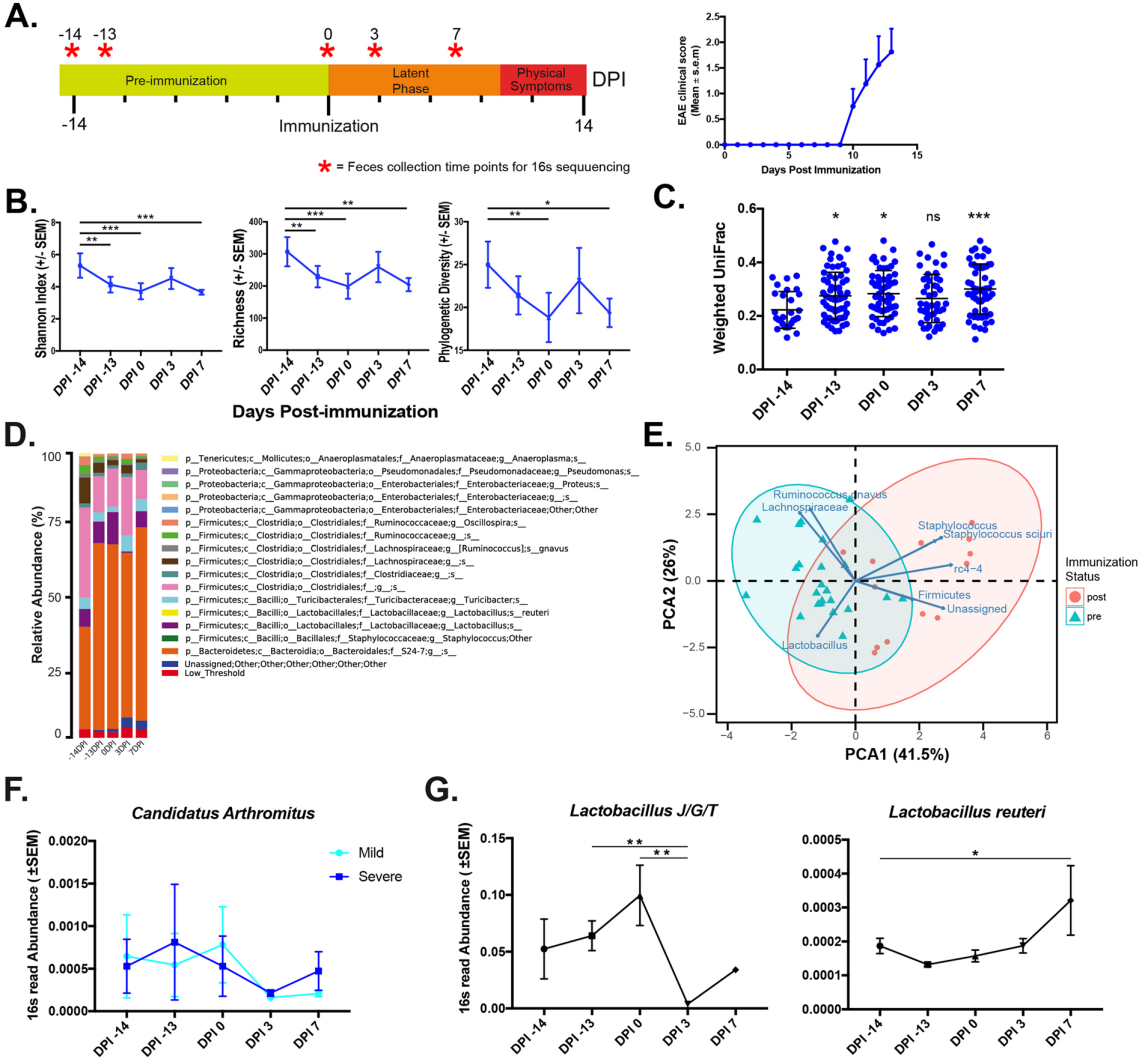
Gut microbe community shows minimal changes during early-phase EAE (**A**). Stool sample collection strategy and EAE clinical scores over time. (**B**) Shannon, richness and phylogenetic diversity over the time course. One-way ANOVA (F = (4,29), *p < 0.05, **p < 0.01, ***p < 0.0001) (**C**). One-way ANOVA with Dunnett’s post-test comparing each group against baseline (DPI -14) weighted UniFrac distances (*p < 0.05, ***p < 0.001). Each circle represents a specific OTU (n = 91). (**D**) Normalized relative abundance stacked bar chart of top 16 microbes over days suggest the greatest deviations in OTU abundances occurred on DPI -14 and DPI 3. In particular, a substantial increase was noted in the order *Clostridia* (pink) on DPI -14 and a loss of the genus *Lactobacillus* (purple) on DPI 3. (**E**) Principle component analysis using significantly altered microbes (p < 0.01) reveal contributors to pre- and post-immunization state separation. (**F**) Two-way ANOVA of *Candidatus arthromitus* reveals no differences in abundance between mildly and severely affected mice (p = 0.86, (**F**) (1, 26) = 0.03139 Dunn’s non-parametric test, Kruskal-Wallis test). (**G**) Analysis of OTUs grouped under the genus *Lactobacillus* (**p < 0.01, Dunn’s non-parametric test, Kruskal-Wallis test = 17.76) and the species Lactobacillus reuteri (*p = 0.0138, Dunn’s non-parametric test, Kruskal-Wallis test = 11.01).

We induced EAE in C57BL/57BL/6J mice (n = 8) with a standard myelin oligodendrocyte glycoprotein (MOG 35–55) adjuvant immunization (Complete Freund’s Adjuvant/Pertussis toxin; CFA/PTX). Mice demonstrated classic symptoms of paralysis and altered balance ten days later, consistent with previous studies (Fig. 1A)^9^. Also consistent with prior studies, these symptoms occurred over a range of severity and varied in their onset timing (Fig. 1A, see Supplementary Table 1 for EAE metrics). We collected fecal specimens from these mice over five time points spanning pre- and post-immunization states prior to disease onset (Fig. 1A). Using these specimens, we first assessed latent period microbial composition changes using 16S rRNA sequencing. After quality filtering (see methods), we observed 2,781 operational taxonomic units (OTUs) from all mice and time points, 91 of which were included at the L6 taxonomic level of the QIIME software output. These 91 OTUs were subsequently used for our subsequent analysis.

Alpha-diversity metrics (within-ecosystem diversity) including Shannon’s diversity index and species richness exhibited no obvious trends over time, although there was significant variability (p < 0.05) with respect to day post immunization (DPI) -14 relative to other days (Fig. 1B). Weighted UniFrac distances (beta-diversity) comparing DPI -14 to later time points also reflected this trend in temporal variation (Fig. 1C). The 16 most abundant microbial taxa, which accounted for approximately 98% of 16S reads, further reflected temporal variation at all time points with respect to DPI -14. For example, we observed an increased in the overall proportion of the family S-24-7 OTUs from 30% on DPI -14 to 66% on DPI -13, and a decrease in the overall proportion of the order Clostridiales-assigned OTUs (12% on DPI -13 to 32% on DPI -14) (Fig. 1D). Taken together, the fairly even variance we observed in the microbiota overall and particularly on DPI -13 through DPI 7 reflect previously described temporal microbial variance in untreated laboratory mouse strains, while DPI -14 is likely reflective of their acclimation period^7,10^.

To preliminarily explore immunization-dependent microbial trends, we considered OTUs which significantly (p < 0.05 ANOVA) and substantially (fold-change > 1.5) between pre- and post-immunization time points, and were represented in >20% of all sampled time points (See Supplementary Table 2 for a list of OTU and their normalized abundance). These criteria yielded nine OTUs, 8/9 (89%) from the phylum Firmicutes. These OTUs exhibited an average fold-change of 2.6. A principle component analysis (PCA) of these data suggested pre- and post-immunization states was partially distinguished by these OTUs (Fig. 1E).

Despite the slight alterations we observed in global microbial community structure during latent period EAE induction, individual taxa could have more relevance to eventual EAE severity. Two taxa were significantly associated with EAE severity scores (p > 0.05, Spearman). The genus *Anaerostipes* exhibited an inverse correlation with EAE severity on DPI 3 (p = 0.049, r^2^ = −0.81, see Supplementary Table 3 for full OTU-EAE correlation statistics) while the family Erysipelotrichaceae was also inversely correlated with EAE (p = 0.03, r^2^ = −0.84), although neither correlation survived multiple-hypothesis correcting FDR, likely due to low replicate numbers and high number of variables (FDR = 0.12, 0.09, respectively). *Anaerostipes*, a butyrate-producing Firmicute, was previously shown to be underrepresented among MS patients^11^. Thus the increased *Anaerostipes* abundance we observed in lower-scoring mice is consistent with previous findings. Previous studies also showed that germ-free (GF) mice colonized with the segmented filamentous bacteria *Candidatus arthromitus* had expanded Th17+ T-cell populations and increased EAE symptoms compared to controls^7,12^. We did not find *C. arthromitus* to be differentially present in mildly (cumulative EAE score 0–4) or severely (cumulative EAE score 5–14) diseased mice. (Fig. 1F). This suggests that while *C. arthromitus* may determine disease presence in a binary fashion, its latent period abundance does not determine subsequent disease severity. Lastly, species and strains within the genus *Lactobacillus* were previously shown to attenuate EAE symptoms, potentially by modulating anti-inflammatory cytokine levels and Th17+ t-cell generation^13–16^, and we found genus-level abundances exhibited a sharp decrease in abundance post-immunization (Fig. 1G). Further characterization of this *Lactobacillus* OTU using BLAST and MEGA7 alignment tools suggested the 16S rRNA reads likely originated from *L. johnsonii*, *taiwanensis*, *gasseri* or a closely related species (SI. 1)^17,18^. However, the related species, *Lactobacillus reuteri*, exhibited a trend towards increased abundance post-immunization (Fig. 1G). These opposing trends echo our prior observation that aggregating OTUs at higher taxonomic levels can obscure contrasting species-level temporal patterns^19^.

Collectively, these results characterize EAE’s latent phase microbiome as having alterations in global structure typical of day-to-day variation, with changes to a small number of taxa post-immunization. These findings align with previous observations that some microbes could impact EAE and also highlight the dearth of microbe-EAE correlations. However, knowing the presence of specific microbes is insufficient to implicate specific host immune response pathways activated in EAE. To more directly assay alterations in host and microbial pathways potentially involved in the latent phase EAE response, we next interrogated the latent phase EAE metaproteome using stool specimens from the same mice.

### Extracellular stool proteins report immunization status

**Figure 2:**
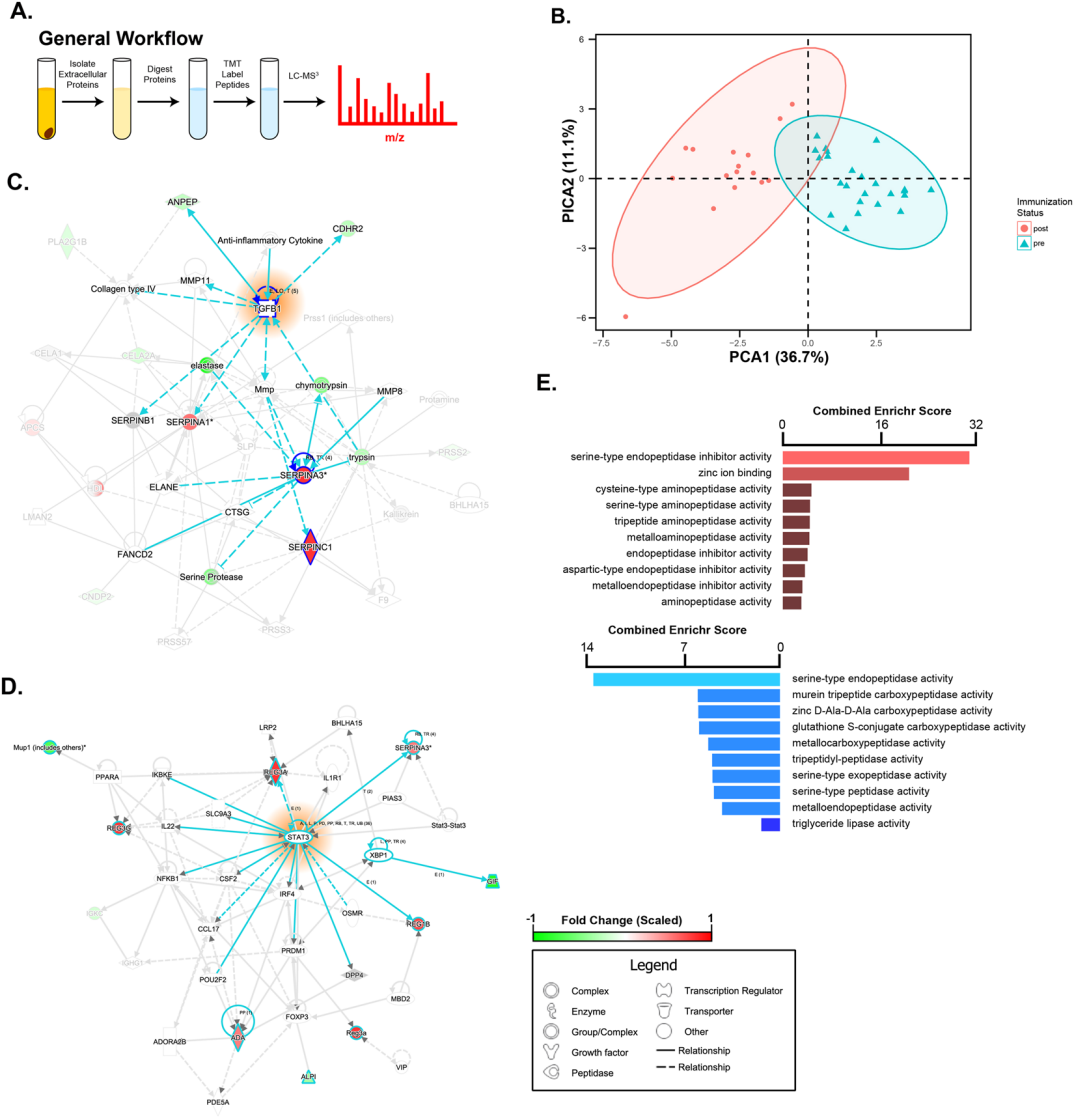
Host stool is a reliable source for tracking extracellular proteins over time in the latent period of EAE. (**A**) Illustration of generalized proteomic workflow. (**B**) PCA generated by proteins significantly altered post-immunization (p < 0.05; F(2,26) ≥ 3.4668) overlaid by confidence interval ovals (CI = 0.95). (**C,D**) An IPA-generated network graph of enriched pathways on DPI 3 (**C**) or DPI 7 (**D**) using statistically altered proteins (p < 0.05, ANOVA) compared to pre-immunization state. Increased abundance is indicated by red shading while green indicates decreased abundance. Turquoise lines indicate pathways highlighted in results section text. Solid lines represent direct associations between detected proteins, while dashed lines represent indirect associations. (**E)** Enrichr-generated gene ontology enrichment analysis (q < 0.01) generated by top 20 proteins with increased and decreased abundance on DPI 3 compared to pre-immunization (average of DPI -14, -13, and 0). The networks were generated through the use of IPA (QIAGEN Inc., https://www.qiagenbioinformatics.com/products/ingenuity-pathway-analysis).

In comparison to 16S rRNA sequencing, metaproteomic analysis of stool can provide both descriptive and functional information about a host’s gut and microbes states over time^8^. Moreover, experimental methods which enrich extracellular proteins from stool can give focus to important immune and antimicrobial interactions^20^. Accordingly, we measured changes to the extracellular fecal metaproteome of mice during the EAE latent period using mass spectrometry. Furthermore, we improved our ability to accurately and efficiently compare mice’s stool proteomes over prior investigations by incorporating a multiplexing quantitative label (tandem mass tag,TMT) into our established workflow (Fig. 2A)^19^. Using this experimental design, we now report 850 quantified proteins (FDR < 0.05) of host (288, 34%) and bacterial (562, 66%) origin (Supplementary Table 4, SI. 2a). Over half (511 total, 218 host, 293 microbes) of these proteins were quantified at every time point from at least one mouse stool sample, suggesting a large portion of detected proteins were stably present (SI. 2b). Despite comprising the majority of protein identifications, microbial protein abundances were on average 1.15 fold (log2) lower than host proteins (SI. 2c).

Applying the same filtering criteria used to select immunization-depended microbe changes to the proteome (p < 0.05, fold-change > 1.5, measurements in >20% of samples). Considering the pre-immunization time points alone indicated proteins which might naturally vary over time regardless of perturbation. We observed 24 proteins (2.8% of total identifications) which met the criteria above, 22 of which were host derived (SI. 3). Two gene ontology classes were significantly enriched (q < 0.01) among these proteins: phospholipase inhibitor activity (GO:0004859, q = 0.0002) which decreased on day post immunization (DPI) -13 compared to DPI -14, and exopeptidase activity (GO:0008238, q = 9.6e-8) increased on DPI 0 compared to DPI -13^21^. These activities are both related to digestion, suggestive of normal dietary processes.

Applying the above criteria to explore differences between pre- (DPI -14, -13, 0) and post-immunization (DPI 3, 7) time points yielded just 39 (5%) proteins (Table 1, SI. 4). Despite this low number, post-immunization states were enriched in several gene ontology classes (Fig. 2B). In particular, host proteins related to antimicrobial activity and protease inhibition were most distinct between these two time periods. These included anti-microbial peptides (AMPs) such as the Regenerating islet-derived protein 3 (REG3, all three isoforms), Serum amyloid P, and Lypd8; and protease inhibitors such as SERPIN A3N, SERPIN A3M, PZP, and SERPIN C1 (Table 1).

**Table 1:** Table of proteins altered during post-immunization.

Accession	Description	p-value	q-value	Log2 Fold change post-immunization
Q9CR35	Chymotrypsinogen B [OS = Mus musculus]	3.18E-08	3.71E-05	−1.389
Q7TPZ8	carboxypeptidase A1 [OS = Mus musculus]	1.60E-07	9.31E-05	−2.353
O09037	Regenerating islet-derived protein 3-alpha [OS = Mus musculus]	2.84E-06	0.00082	3.962
Q91WP6	Serine protease inhibitor A3N [OS = Mus musculus]	2.29E-06	0.00082	2.628
P05208	Chymotrypsin-like elastase family member 2 A [OS = Mus musculus]	1.62E-05	0.0037	−2.511
P35230	Regenerating islet-derived protein 3-beta [OS = Mus musculus]	2.37E-05	0.0046	2.726
P52787	Gastric intrinsic factor [OS = Mus musculus]	8.19E-05	0.012	−2.555
O09049	Regenerating islet-derived protein 3-gamma [OS = Mus musculus]	0.00097	0.12	2.397
Q91WG0	Acylcarnitine hydrolase [OS = Mus musculus]	0.0012	0.14	−1.873
P12246	Serum amyloid P-component [OS = Mus musculus]	0.0030	0.27	1.410
Q03734	Serine protease inhibitor A3M [OS = Mus musculus]	0.0052	0.40	1.749
Q9CQ52	Chymotrypsin-like elastase family member 3B [OS = Mus musculus]	0.0052	0.40	−1.339
P32261	Antithrombin-III [OS = Mus musculus]	0.0060	0.44	1.479
A2AEP0	Odorant-binding protein 1b [OS = Mus musculus]	0.0086	0.51	−1.601
Q61838	Pregnancy zone protein [OS = Mus musculus]	0.0087	0.51	1.874
P09528	Ferritin heavy chain [OS = Mus musculus]	0.0099	0.55	−1.033
P07758	Alpha-1-antitrypsin 1-1 [OS = Mus musculus]	0.011	0.60	1.399
Q9Z0Y2	Phospholipase A2 [OS = Mus musculus]	0.014	0.70	−1.355
P11438	Lysosome-associated membrane glycoprotein 1 [OS = Mus musculus]	0.015	0.72	−1.045
A2BIM8	major urinary protein 18 [OS = Mus musculus]	0.016	0.73	−1.720
HMPREF9402_0233	glyceraldehyde-3-phosphate dehydrogenase, type I [Turicibacter sp. HGF1]	0.016	0.73	1.164
P01644	Ig kappa chain V-V region HP R16.7 [OS = Mus musculus]	0.023	0.96	−1.1957
CUW_1151	N-acetylmuramoyl-L-alanine amidase [Turicibacter sanguinis PC909]	0.028	0.99	1.069
P11589	Major urinary protein 2 [OS = Mus musculus]	0.040	0.99	−1.966
P22599	Alpha-1-antitrypsin 1-2 [OS = Mus musculus]	0.036	0.99	1.118
Q00897	Alpha-1-antitrypsin 1–4 [OS = Mus musculus]	0.039	0.99	1.070
Q91X79	Chymotrypsin-like elastase family member 1 [OS = Mus musculus]	0.035	0.99	−1.069
Q9D7S0	Ly6/PLAUR domain-containing protein 8 [OS = Mus musculus]	0.037	0.99	1.1380

Post-immunization also contains fold-change statistic due to its two-state comparison (pre- vs. post-immunization).

We next considered potential signaling pathways enriched post-immunization. Given the general stability of the pre-immunization time points in all mice, we selected DPI -14 as the reference time point for further comparisons. Ingenuity Pathway Analysis (IPA) of significantly altered protein abundances (p < 0.05) on DPI 3 compared to DPI -14 implicated the anti-inflammatory cytokine TGF-ß as a central signaling node, although the cytokine itself was not identified in the dataset (Fig. 2C). Protease inhibitors, as a class were a dominant feature of this protein network. Indeed, further analysis of the top 20 proteins with changed abundance on DPI 3 confirmed a dramatic depletion of protease-activity related proteins while simultaneously reporting increased protease inhibitors (q < 0.01), supporting the IPA findings (Fig. 2E). This evidence suggests mice upregulate a subset of acute-phase proteins related to inflammation reduction during early latent period.

In contrast, IPA of the 34 proteins with significantly altered abundances on DPI 7 relative to DPI -14 pointed toward increased activity in STAT3, a pro-inflammatory transcription factor central to several intestinal disorders, or a related pathway (Fig. 2D)^22^. Reg3 AMPs were major contributors to the enrichment of this inflammatory network and were notably higher on DPI 7 compared to DPI 3 (SI. 5). This finding is bolstered by previous research suggesting STAT3 activation was necessary for antimicrobial peptide expression^23^. Together, these data suggest that latent period extracellular stool proteins reflect at least two host-response components that are highly targeted and coincide with latent period EAE: an anti-inflammatory phase marked by increased protease inhibitors (DPI 3), and an inflammation-driven phase skewed toward microbial control (DPI 7).

### Immunization alters host gastrointestinal protease and protease inhibitor levels

The ontology analysis above suggested that increased protease inhibitor abundance and concomitant decrease in protease abundance (Fig. 2E) could be endpoints of an inflammation response induced by the experimental EAE immunization. Alternatively, they could reflect separate unrelated stimuli such as feeding. Proteases within the intestinal lumen canonically break down dietary proteins, but can also alter epithelial integrity, thereby regulating the intestinal environment and corresponding immune responses^24–27^. In this context, host-expressed protease inhibitors play protective roles, potentially by limiting epithelial damage proteases might otherwise inflict. To further evaluate possible coordination between these antagonistic protein groups, we quantified all 29 proteases and 14 protease inhibitors from the experiment described in Fig. 2, of which 83% and 100% were host-derived, respectively (Supplementary Table 5). All identified protease inhibitors were members of the I4 inhibitor family^28^, which target S1 serine proteases including trypsin, chymotrypsin, mast cell protease, and granzymes. Seven (24%) of the proteases we identified belonged to the S1 family. However, the remaining protease families were made up of just 1–2 (<10%) proteases, while five (17%) had unknown family associations.

**Figure 3:**
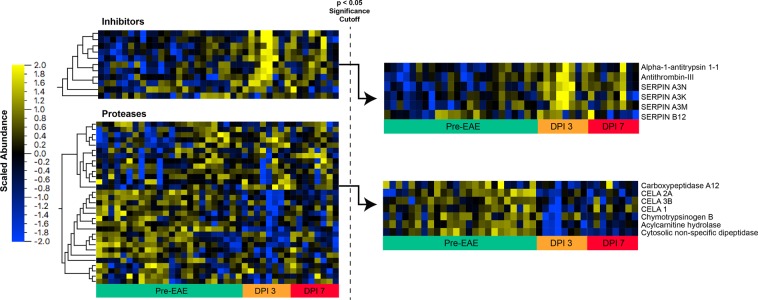
Immunization results in alterations in host gastrointestinal protease and protease inhibitor abundance. Normalized and scaled heatmap of all protease and inhibitor reporter abundance over the course of the study, followed by a subset from both groups consisting of proteins found significantly (p < 0.05 ANOVA; F(4,35) ≥ 2.6415) altered post-immunization.

Despite the diversity in observed protease families, we found that proteases and protease inhibitors which significantly changed before and after immunization (p < 0.05) did so in a roughly mutually exclusive fashion (Fig. 3). Whereas significantly altered proteases decreased 1.6–28 fold (log2 0.7–4.8) upon immunization, significantly altered protease inhibitors increased 1.5- to 64-fold (log2 0.6–6.0). It is noteworthy that 71% of decreased proteases were host-expressed S1 serine proteases, consistent with host-expressed protease inhibitors targeting host-expressed proteases. This inverse relationship was particularly pronounced on DPI 3, suggesting coordinated protease and protease inhibitor expression early in the latent period.

Despite the roughly binary pattern exhibited by a subset of proteases and protease inhibitors, we noted substantial variation in protease inhibitor abundances across days and mice. As mentioned above, we also noted that mice demonstrated a wide range of clinical severity (cumulative score range = 0.5–13, see Supplementary Table 1). Since protease activities have known roles in regulating innate immune and gut-permeability responses, we next examined whether the protease and protease inhibitor variations we observed corresponded with EAE disease severity^29^.

### Protease inhibitor abundances foreshadow EAE symptom severity

**Figure 4:**
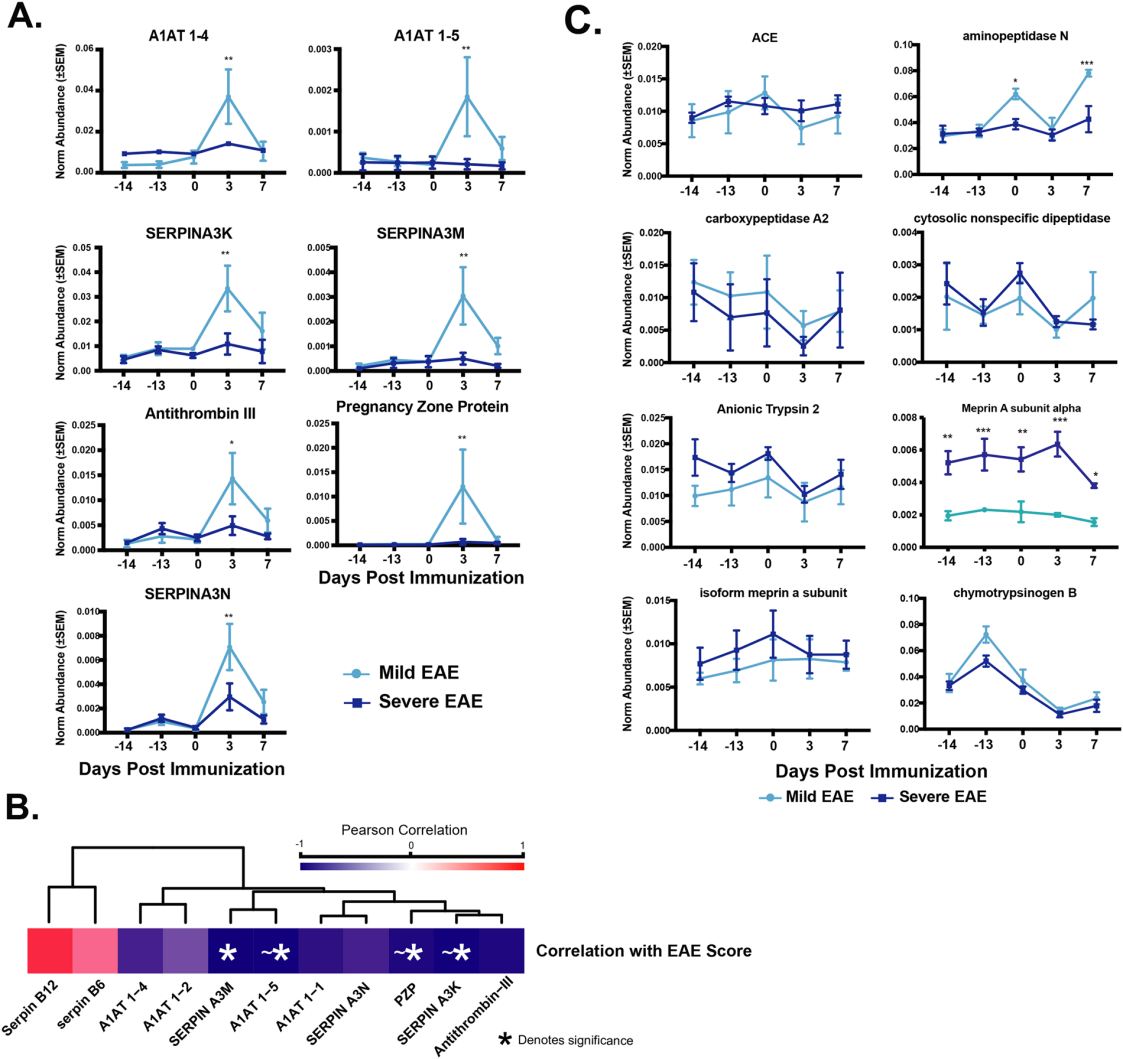
Protease inhibitor abundance is differentially present in mild and severe EAE mice prior to disease onset, while proteases vary minimally. (**A**) Two-way ANOVA (n = 4 mice per group) of selected protease abundance in mild and severely scored EAE mice over the course of the study, asterisks denote significance (Dunn’s test corrected *p ≤ 0.05, **p ≤ 0.01, ***p ≤ 0.001). The protease subset selected is representative of the overall trend in protease abundance. (**B**) Pearson correlation analysis of protease inhibitor abundance and EAE cumulative severity. SERPIN A3M is noted as being significantly correlated, while several others (denoted as ~*) nearly reached significance (p ≤ 0.1, see Supplementary Table 6). Higher EAE score is indicative of greater disease severity (**C**). Two-way ANOVA of protease inhibitor abundance in mild and severely score EAE mice over time suggested significance between mild and severely affected mice specifically on DPI 3 (Bonferroni corrected, *p ≤ 0.05, **p ≤ 0.01, ***p ≤ 0.001; for complete statistics see Supplementary Table 8).

Binning the mouse cohort into mild and severe EAE groups as in Figure 1F revealed that 71% of all protease inhibitors measured here were 1.4–8.6 fold (log2 0.5–3.1) greater on DPI 3 relative to pre-immunization time points in mildly affected mice compared to their severe counterparts (Fig. 4A). Accordingly, these protease inhibitor abundances were inversely correlated with disease severity. However, we note that as with the microbe-EAE correlation analysis (Supplementary Table 3), many protein-EAE correlations did not exceed a multiple hypothesis corrected FDR threshold of 0.05, due to the low number of replicates per treatment group and large number of considered proteins (Fig. 4B, see Supplementary Table 6 for complete statistics).

Protease abundance corresponded with mild and severely afflicted mice to a much lesser degree than the protease inhibitors (Fig. 4C). One notable outlier was meprin-α, a M12-family metalloendopeptidase, which was significantly increased (2.4–3.1 fold increase, p < 0.05) in all mice which developed severe EAE at all pre- and post-immunization time points, supporting a notion that pre-immunization states could influence eventual disease outcomes. In line with this, meprin-α abundance, but not an isoform detected, was the only protease significantly (p = 0.02, r^2^ = 0.8) correlated with EAE cumulative severity score, although several other proteases also showed trends towards significance (Supplementary Table 6). Together, these data suggest that extracellular host protease levels largely did not coincide with severity, despite their known roles in gut-related immunity modulation. In contrast, the inverse relationship between protease inhibitors’ latent period expressionand eventual EAE symptoms was strong and consistent across this protein class. This suggests that regardless a proteolytic activity’s source during the latent period, its control can be achieved through the coordinated expression of multiple protease inhibitors. This expression could modulate, or at least forecast eventual disease severity.

### Protease inhibitors correlate to specific microbes in early latent period EAE

Microbial influence over host systems have been studied in many contexts including the activation of the kallikrein-kinin system^25,30^. However, these associations remain largely unexplored in the case of protease inhibitors and more specifically, during EAE’s latent period. Identifying potential associations between proteases, protease inhibitors, and OTUs would be a critical first step in establishing a proteolysis-focused host-microbiome interaction model in EAE.

**Figure 5:**
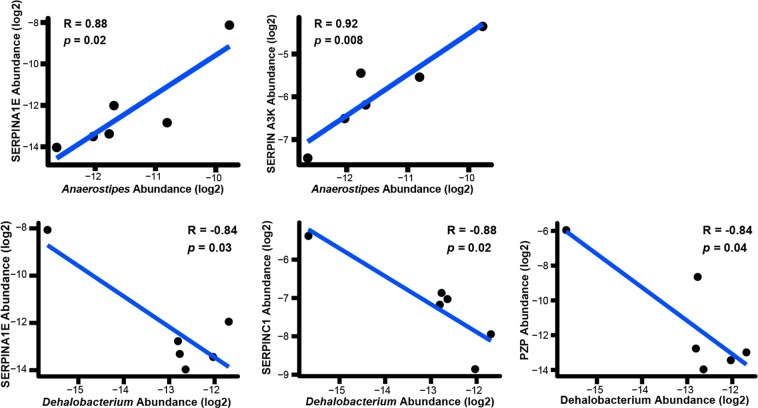
Protease inhibitor abundance correlates with specific microbe abundance on DPI 3. Scatterplot of log2 normalized *Anaerostipes* and *Dehalobacterium* abundance versus log2 normalized protease inhibitor abundance exhibited significant correlations (p ≤ 0.05) on DPI 3. Each dot represents a different mouse (n = 6 due to 16s sample unavailability).

To this end, we correlated protease and protease inhibitor abundances with OTU levels, focusing on DPI 3. The resulting 54 × 54 Pearson correlation matrix (SI. 6) implicated 85 significant (p < 0.05) correlations, including 21 protease-OTU pairs and 9 protease inhibitor-OTU pairs (see Supplementary Table 7). For example, the largely uncharacterized genus *rc4-4* (family *Peptococcaceae*) was positively correlated with the inhibitor SERPINB12 (r^2^ = 0.92, p < 0.05) and negatively correlated (r^2^ = −0.84, p < 0.05) with SERPINA1A. While little is known of *rc4-4*, previous studies have revealed its enrichment along with another *Peptococcaceae* family member, *Dehalobacterium*, in the stools of MS patients^31^. Our findings also revealed a significant (p < 0.05, r^2^ −0.88 to −0.84) negative correlation between *Dehalobacterium* and three protease inhibitors (Fig. 5), but no significant correlation with proteases (not shown). Contradicting reports have found the genus *Anaerostipes*, which shares the order Clostridiales with *rc4-4* and *Dehalobacterium*, to be both enriched and depleted in MS patient stool^5,31^. Here, *Anaerostipes* was positively correlated with the abundance both SERPINA1E and SERPINA3K and, interestingly, several proteases (Fig. 5). Of note, only *Anaerostipes* was positively associated with both protease inhibitors and negatively with EAE. This is a result of weak correlations in the overlapping inhibitor-OTU-EAE correlations. These data further support the notion that specific taxa may have different functional impacts on host – impacts which could involve the induction or inhibition of proteolysis.

### Adjuvant alone, but not vancomycin, increased protease inhibitor abundance

In addition to the inflammation-driven proteolysis regulation pathways explored above, the gene ontology analysis presented in Fig. 2 suggested a second, antimicrobial expression program engaged by the EAE immunization prior to symptom onset. These phenomena may be linked in multiple ways. As the previous section suggested, specific microbes could be associated with increased or decreased latent period levels of protease inhibitors, among other proteins. As such, we wished to directly test the dependency of latent period protease inhibitor levels on a subset microbes necessary for EAE induction. Antibiotic administration has been widely reported as a suppressor of EAE’s neurologic symptoms^32–34^. Less understood is antibiotic’s ability to alter the gut immune response^35,36^. Because a wide range of microbe-interacting proteins can be readily detected with our stool-based assay, we hypothesized mice protected from EAE symptoms by antibiotic treatment could reveal microbe-dependent host responses important for EAE’s progression. Specifically, if the increased protease inhibitor abundance described in Figs 2–4 depended on microbes, then antibiotic administration should reduce DPI 3 protease inhibitor levels. Alternatively, if the microbes associated with the increased inhibitor levels were resistant to antibiotics, inhibitor levels should remain high.

**Figure 6:**
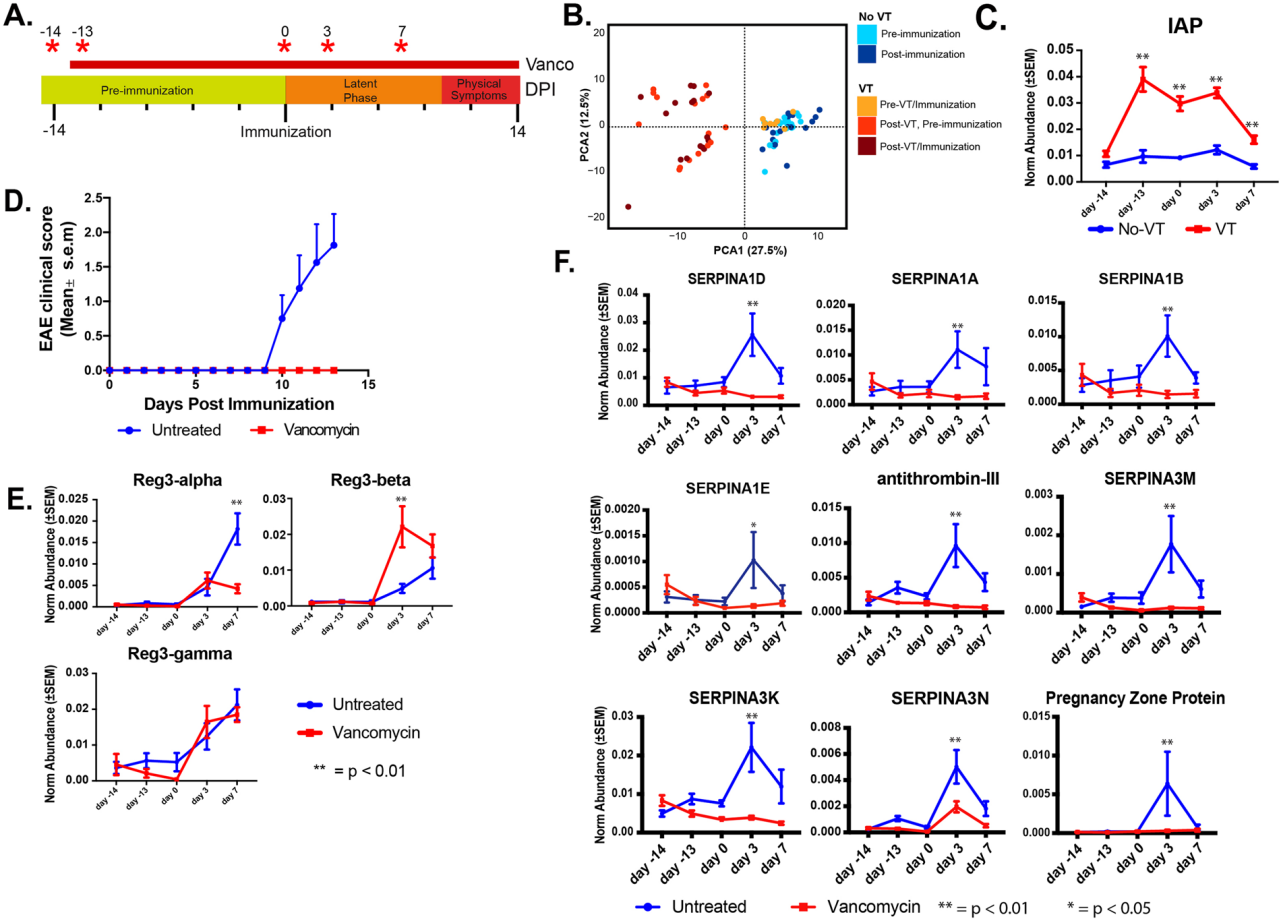
Vancomycin alters stool proteome and reduces protease inhibitor abundance. (**A**) Timeline of vancomycin treatment and stool collection schedule. (**B**) PCA of Vancomycin and untreated groups over the time course. Graph was generated from the subset of proteins (N = 177) that differentiated each day and group using statistically significant (two-way ANOVA, Bonferroni correction p < 0.05) (**C**). Two-way ANOVA of intestinal alkaline phosphatase levels over the time course (Bonferroni’s correction, **p < 0.01; F (4, 56) = 22.35). (**D**) Mean clinical score over course of study. The non-antibiotic treated group exhibited significantly (two-way ANOVA, Bonferroni correction p < 0.05) increased mean scores compared to their vancomycin-treated counterpart at every point past DPI 9. (**E**) Two-way ANOVA comparison between VT and untreated mice of Reg3-family AMP abundance over time (two-way ANOVA, Bonferroni correction p < 0.05). (**F**) Time course comparison of selected protease inhibitor abundance found in vancomycin-treated and non-treated mice stool (two-way ANOVA, Bonferroni correction p < 0.05; See Supplementary Table 8 for complete statistics).

Antibiotics can have idiosyncratic effects on the host when systemically present^37^. To address this, we orally administered vancomycin to a separate cohort of mice (n = 8) on DPI -14 and characterized their 16S rRNA and stool proteome as previously described (Fig. 6A). Since vancomycin is poorly absorbed by the intestine, its action should be restricted to the gut microbiota, thereby minimizing confounding systemic effects. Moreover, vancomycin has previous been used as part of antibiotic cocktails administered to mice during EAE. We found that vancomycin pre-treatment (VT) entirely abrogated EAE induction over the fourteen days following standard EAE immunization (Fig. 6D). As expected, the antibiotic was a major perturbation to the gut microbiota, as measured by decreased phylogenetic diversity, richness, and Shannon index metrics (SI. 7a). A PCA generated from significantly altered (32/91 OTUs, p < 0.05) OTUs revealed a complete separation between pre-VT and post-VT states (SI. 7b). Pre- and post-VT weighted UniFrac distances also indicated increased phylogenetic distance between VT and non-VT groups (SI. 7d). The top 16 most abundant OTUs also mirrored this trend, revealing a depletion of the S24-7 family (47% of all 16s reads on DPI -14 to 0.2% on DPI -13) and unassigned members of the Clostroidales order (22% of 16s reads on DPI -14 to 2% on DPI -13). In parallel, VT also coincided with the emergence of the Enterobacteriaceae family (0.002% of 16S reads on DPI -14 to 55% on DPI -13) as the dominant OTU (SI. 7d). VT also significantly and durably reduced the abundances of the genera *Anaerostipes* and *Dehalobacterium* to undetectable levels (SI. 11).

Consistent with our previous proteomic analysis of antibiotic-exposed mice, stool protein profiles of VT mice remained distinct from their pre-VT state and from non-VT mice throughout the pre-immunization and latent period time course (Fig. 6B), mirroring changes in OTU abundances (SI. 7)^19^. Proteins upstream and downstream of the STAT-3 pathway such as CEAMCAM1, CNDP2, and SERPINB1 were downregulated following VT (SI. 8a). This suggests VT-sensitive microbes are associated with alterations in STAT-3 inflammatory related pathways (Fig. 2D). In addition to decreased inflammation pathways, we found a significant increase in intestinal alkaline phosphatase (IAP) after VT, which persisted through DPI 7. In contrast, IAP remained constantly low in non-VT immunized mice (Fig. 6C). IAP, an intestinal brush border enzyme, was previously shown to impede the translocation of microbes across the intestinal barrier^38^, suggesting one possible mechanism for antibiotic-mediated protection. Impaired barrier integrity has previously been noted in EAE mice^39^, supporting this notion.

Just as VT mice displayed no outward signs of EAE’s classic physical impairments throughout the course of the study (Fig. 6D), they demonstrated only modest protein responses to EAE immunization. Little overlap was apparent from the protein networks induced by immunization with and without VT pre-treatment (Fig. 2C,D, SI. 8b). For example, when compared to pre-immunization time points, post-immunization levels of AMPs were elevated in both VT and non-VT mice. However, we recorded significantly higher levels of Reg3-ß from VT mice on DPI 3 compared to non-VT mice (mean fold change = 4.5, p < 0.0001; Fig. 6E). In contrast, Reg3-α levels were equal in both groups on DPI 3, but significantly higher on DPI 7 in non-VT mice compared to VT counterparts (mean fold change = 4.2, p < 0.0001; Fig. 6E). Reg3-γ increased at a similar rate in both untreated and VT mice (Fig. 6E). These data suggest that despite exhibiting no physical symptoms, immunization triggers an active-but-alternative antimicrobial response in mice pre-treated with VT. These responses include an alternate expression profile of the three Reg3 isoforms.

In line with an alternative post-immunization host response, the majority of protease inhibitors remained unchanged or even decreased over the course of the study in VT mice (Fig. 6F). Indeed, protease inhibitor levels in VT mice were significantly (p < 0.05, ANOVA) decreased when compared to non-VT mice at DPI 3 and 7 (Fig. 6F). Protease abundances were altered after VT (DPI -13) and immunization (DPI 3) (SI. 9). Together these findings further support increased protease inhibitor levels in non-VT mice as being dependent on the presence of byproduct of VT-sensitive microbes while simultaneously suggesting that altering microbial communities also shifts the antimicrobial response compared to non-VT mice.

These data suggests post-immunization protease inhibitor levels are responsive to a subset of intestinal microbes. However, the CFA/PTX adjuvant required for EAE induction in this model could also influence protease inhibitor responses. To better understand the adjuvant’s contribution to the protease inhibitor responses we measured, we treated six mice with CFA/PTX without adding MOG, and collected stool from DPI 0 and 3. Stool proteins quantified by label-free methods indicate that CFA/PTX alone significantly (p < 0.05) increased protease inhibitor levels on DPI 3 (SI. 10). These results support systemic adjuvant administration as promoting microbe-dependent protease inhibitor increases, as measured from stool.

In sum, CFA/PTX adjuvant increased protease inhibitor levels regardless of MOG presence. Additionally, abundance changes in stool proteins suggest VT induces an alternative host response to immunization, possibly by altering inflammatory pathway signaling and microbial translocation potential. This implicates vancomycin-sensitive microbes in the priming or aggravation of immune system components, driving EAE to manifest.

## Discussion

Defining pre-symptomatic molecular determinants of disease is critical for developing new strategies for early detection, prevention, and therapies^1–3^. Thus, disease models with defined and reproducible latent periods are valuable tools for identifying such molecular determinants. Further, they can help refine strategies for elucidating and prioritizing those which correspond with eventual symptoms. This study aimed to elucidate stool proteins which shape host-microbe interactions during the latent period of EAE induction, a model of relapsing-remitting MS. This first-reported stool proteome of the EAE model identified several protein classes and their associated pathways in the latent period that could be the focus of future targeted prophylactic or therapeutic studies.

First, we showed the extracellular stool proteome readily differentiated pre- and post-immunization states at least as well as their underlying microbiota. Unlike the microbial enumeration, we showed that host proteins driving this differentiation provided functional evidence relevant to EAE etiology. For example, we noted AMPs were upregulated post-immunization regardless of antibiotic treatment. This suggests that regardless of whether eventual external EAE symptoms manifested, the immunization cocktail consistently triggered antimicrobial responses during latent period. We further note only modest changes to the microbiome between mice immunized adjuvant alone, versus the CFA/PTX adjuvant with MOG. Considering this, it is unlikely that this antigen substantially contributed to any effects we could measure from stool. Together, these results raise questions as whether the CFA/PTX adjuvant alone is capable of modulating the gut microbiome, and whether such a change could sensitize animals to a broader array of autoimmune disease models.

We further showed that antibiotic exposure had a pronounced effect on protein expression. Specifically, latent period AMP expression patterns only partially agreed between VT and non-VT mice. While both treatment groups demonstrated increased Reg3-γ levels following immunization, VT mice showed increased Reg3-β on DPI 3 relative to non-VT mice, while non-VT mice showed increased Reg3-α on DPI 7. These discrepancies in Reg3 AMP isoforms could reflect the major differences in microbe community structures each group at the time of immunization, with a greater proportion of gram-negative microbes present in VT mice. However, Reg3-β’s noted role in limiting translocation of gram-negative microbes across the intestinal barrier^40^ should be considered when interpreting this result. Given that molecular mimicry in EAE and MS is one prevalent etiological theory, the protection from ensuing microbe-induced inflammation could reduce systemic levels of potential MOG molecular mimics^41^.

We also found that intestinal serine protease inhibitor levels increased with immunization in a manner that inversely correlated with EAE severity. This suggests that members of this protease inhibitor class could antagonize pathogenic host-host or host-microbe interactions. In support of this, oral administration of Bowman-Birk or PETIR001 serine protease inhibitors were previously shown to ameliorate EAE’s classic neurological symptoms^42–44^. Given the important role protease inhibitors have in tempering systemic immune system responses, as well as self-tolerance, our findings are not entirely surprising^45^. For example, the balance between trypsin and alpha-1-antitrypsin (SERPINA1) is well-studied in the case of viral infection and cystic fibrosis where protease-inhibitor levels protect airways from an aberrant inflammatory response^46,47^. However, the consequences of altering the levels of any single specific endogenous protease inhibitor (either in the stool or peripherally) prior to EAE onset are currently unknown. Our study supports a model in which the host expresses a variety of serine protease inhibitors into the GI lumen to protect the surrounding epithelium from otherwise aberrant and toxic proteolytic activity. The upregulation of protease inhibitors, and thus the applicability of these findings, during flare and remission states in MS have yet to be determined.

Based on prior studies that demonstrated the EAE model’s dependence on the microbiota, we suspected antibiotic treatment would reveal protective proteomic mechanisms, and given the increased inhibitor abundance in non-VT mice, we predicted a similar increase in VT mice. Surprisingly, we found that VT, which prevented EAE symptoms, did not alter protease inhibitor levels, which our data suggested was protective in non-VT mice. These results raise several questions about the biological underpinnings driving these findings and the potential compensatory role of protease inhibitors in EAE. One possible mechanism explaining the discrepancy between VT and non-VT mice is that specific VT-sensitive microbes (or subset of the microbiome) trigger the release of host-expressed protease inhibitors. The magnitude of their expression, at least in part could then dictate the subsequent degree of EAE severity. When these triggering microbes are removed by VT, protease inhibitor levels remain at basal levels. Alternatively, an increase microbial debris resulting from VT could also trigger an increase in “protective” proteins (e.g. IAP and AMPs) that lead to increased epithelial integrity, decrease immune system “reactivity”, and control microbial growth. Supporting this, IAP has previously been described in several capacities as anti-inflammatory through its regulation of Th17 + t-cell generating pathways – important to EAE pathology – and its role in gut epithelium integrity^48^. Stated simply, it is possible that VT’s action prevents immune system activation prior to EAE induction, thus preventing the need for increased protease inhibitor production and secretion by the host during EAE. This suggests that protease inhibitor expression can be effective in modulating the inflammation necessary for EAE induction; however, this protection is less effective than preventing any microbe-dependent inflammation in the first place. To confirm any of these findings, future studies should examine the correspondence between protease inhibitor expression and immune cell phenotype changes.

Previous findings suggest VT also reduces lamina propria-resident Th17+ T-cell populations in mice^35,36^. These findings also suggested members of the vancomycin sensitive Cytophaga-Flavobacteria-Bacteroides taxonomic group (currently known as the phylum Bacteroidetes) strongly correlated with Th17+ T-cell levels. This indicates a subpopulation of vancomycin-sensitive microbes affect Th17+ T-cell generation. However, the mechanisms by which these microbes directly or indirectly influence host immunity remain unclear. Prior work has showed the Th17-dependent differentiation is influenced by the balance of proteases (cathepsin B/L) and protease inhibitors (SERPNB1)^49–51^. This suggests one possible mechanism microbes could use to influence the host immune state is protease activity. In support of this, previous research reported that proteases are often preferentially packed into outer membrane vesicles generated by the genus *Bacteroides*, a genus within the Bacteroidetes phylum^52^. However, given that a majority of proteases we detected were host derived, more work must be done to isolate microbial proteases if this hypothesis is to be substantiated. While our study did not attempt to associate EAE proteome signatures to immune cell populations levels, our findings along with previous evidence point to specific immune cell activation which is dependent on latent period protease and protease inhibitor levels in the gut.

In addition to the role proteases have in T-cell differentiation, experimental colitis and other research models have indicated aberrant protease activity contributes to the loss of intestinal barrier integrity and to chronic inflammation^53,54^. Decreased intestinal barrier integrity has also been shown in EAE^39^. One mechanism by which protease-protease inhibitor interactions influence EAE could involve protease activated receptors (PARs). Receptors in this family such as PAR2 have well-noted roles in gut epithelium integrity and are also present throughout the vasculature and cells residing in the CNS^27,55^. Prior research has noted an upregulation of PAR2 on astrocytes of EAE mice and MS patients, suggesting these cells may be more sensitive to perturbations in trypsin-like protease activity compared to healthy controls^56^. Our study supports this notion in that all protease inhibitors we identified as increased on DPI 3 belonged to the I4 family – a family that inhibits known PAR activators such as trypsin and tryptase.

Remarkably, the abundance of several proteases remained largely unchanged over the experimental time course, while others were severely decreased. As such, the increased protease inhibitor levels we observed could reflect a response to increased protease activity. This increase in activity may result from active proteases already present or an increase in protease abundance from unidentified source such as bacteria and unicellular eukaryotes, or some combination thereof. Unchecked by protease inhibitors, such increased proteolytic activity could lead to increased microbial infiltration via weakened epithelial junctions, leading to sustained inflammation. One avenue to test this hypothesis is through characterizing the stool of germ-free animals inoculated with representative microbial community members suspected of EAE exacerbation. Using this approach along with genetic manipulation of known proteases within this selected microbial community could further elucidate the role of specific proteases in EAE, as well as the inhibitor(s) responsible for limiting their activity. However, in order to undertake the proposed line of experimentation, proteases and inhibitors must be identified from an extremely complex mixture of proteins originating from every taxonomic kingdom. Despite identifying 842 microbial proteins (including proteins only found in VT mice), which comprised 72% of our dataset, we were only able to detect five microbial proteases, a result likely due to several factors such as mass spectrometer dynamic range and database sequence exclusion^8,57^. Additionally, a lack of accurate sequence annotation also likely hampered effective protease identification. Indeed, our search identified 135 (16%) microbial proteins with no attached annotation. As more microbes are sequenced and protein functions are inferred, this limitation will likely be lessened.

Accurate identification of both proteins and microbes will be especially important for translating these results into more clinically tractable settings. It is currently unknown whether similar increases in gut-specific protease activity or inhibitor production are present in MS patients immediately prior to or during a flare, or more importantly, if they play a primary or compensatory role in MS severity modulation. However, previous findings in humans have hinted at their role in plasma^58^. Given our ability to detect these events in animal models, future studies should focus on longitudinal collection of stool with the goal of characterizing the stool proteome in healthy, pre-flare, and flare conditions. Currently, we are attempting to collect such a cohort. Such a study could lead to the development of convenient at-home diagnostic tests for increased protease activity or inhibitor levels.

Overall, this study both expands and reinforces our understanding of the diverse ways hosts and microbes interact during health and disease and are summarized in SI. 12. By leveraging multi-omic technology and time-tested physiological phenotype measurements, we were able to elucidate novel host-microbe interactions with the potential to impact future research and human health.

## Methods

### Mice

C57BL/6J female mice were obtained from The Jackson Laboratory and kept in a specific pathogen-free facility at the Harvard Institute of Medicine (n = 8 for MOG/CFA/PTX and VT experiments, n = 6 for CFA/PTX only, randomly assigned) on a 12 hour light/dark cycle. Mice were all 8–10 weeks of age and cohoused, four mice from same experimental condition per cage. Mice were fed an ad-libitum diet of Picolab Rodent Diet 5053 and distilled water without added preservatives (provided by BWH animal facility). All animal experiments described in this paper were approved by the Institutional Animal Care and Use Committee (IACUC) at Harvard Medical School and carried out in accordance with those approved animal experiment guidelines. Mice receiving vancomycin were housed separately from non-vancomycin treated mice. Precise mouse weight was not recorded, however signs of weight loss were assessed by lab facilities each day and no weight loss was noted during the latent period with or without vancomycin administration.

### EAE induction and treatments

EAE was induced in mice by subcutaneous immunization with 100 ug MOG 35–55 peptide emulsified in complete Freund’s adjuvant (CFA, Difco Laboratories) per mouse, followed by intraperitoneal administration of 150 ng pertussis toxin (PTX, Millipore) on days 0 and 2 as previously described^59^. Clinical signs of EAE were assessed (not blinded) at the same time each afternoon according to the following score: 0, no signs of disease; 1, loss of tone in the tail; 2, hind limb paresis; 3, hind limb paralysis; 4, tetraplegia; 5 moribund^59^. Scoring was not blinded to experimental condition. For VT mice, Vancomycin was added to the water bottle at a concentration of 0.5 g/L (Thermo Fischer) on DPI -14 after stool pellets were collected. All control mice receiving CFA/PTX without MOG 35–55 were subjected to the same protocol as experimental mice.

### 16S rRNA microbial community profiling

Fecal DNA was isolated (MoBio PowerLyzer PowerSoil Kit) and the V4 region of the bacterial 16S rRNA gene was amplified using barcoded forward primers (515F, 806R) from the Earth Microbiome Project and http://www.earthmicrobiome.org/protocols-and-standards/16S/ ^60^. Samples were sequenced by paired end 150 base pair reads at the Harvard Medical School Biopolymers Facility using the MiSeq platform (Illumina). Sequence quality was evaluated with FastQC (http://www.bioinformatics.babraham.ac.uk/projects/fastqc/). The median Phred quality scores were above Q30. Downstream analysis was performed in QIIME (Qualitative Insights into Microbial Ecoloy)^61^. Sequences were de-multiplexed and quality filtered in which reads are truncated if two consecutive bases fall below a quality score of Q20 (1% error), and reads that are <75% of full length are discarded^62^. OTUs were picked using the open reference method sumaclust (http://metabarcoding.org/sumatra) and sortmeRNA^63^. Taxonomy was determined from the Greengenes database (13.8 release; http://greengenes.secondgenome.com) using a 97% similarity threshold. Diversity metrics were calculated from a rarefied table at an even depth of 1000 reads per sample. Alpha diversity was calculated with the phylogentic diversity whole tree, Shannon’s diversity, richness metrics, and beta-diversity were calculated using weighted and unweighted UniFrac distances. Further identification of the top 10 Lactobacillus OTUs analysis was performed using BLAST^18^ and MEGA7: Molecular Evolutionary Genetics Analysis version 7.0 (MEGA)^17^. Briefly, phylogenetic relationships of the closest BLAST matches as well as other reference bacteria were use to generate a phylogenetic tree in MEGA using the neighbor joining method.

### Isolation of stool proteins

Multiple stool pellets from each specific mouse were freshly collected directly into an 2 mL Eppendorf tube each day, immediately flash frozen and stored in a −80 C freezer. Pellets were collected at approximately the same time each day. Pellets from all time points were collected, an appropriate number of these pellets (1–2 pellets) were shipped on dry ice for further handling in the Elias lab. Upon arrival, samples were stored in −80c freezer. All samples were prepared in a blinded, randomized order and processed as previously described in^20^. Briefly, sample pellets were disrupted using 500 uL urea lysis buffer (8 M urea, 100 mM NaCl, 25 mM Tris, pH 8.2) supplemented with Roche cOmplete mini protease inhibitor (04693159001 ROCHE) by vortexing. After pellet resuspension, insoluble material was pelleted at 2500× g for 10 minutes at 4 °C and the resulting supernatant was subjected to ultracentrifugation (35000 RPM [avg. RCF 43492] for 30 min. at 4C, Beckman-Coulter Optima Ultracentrifuge, rotor TLA120.2) to remove bacteria and small debris. The ultracentrifuge supernatant was subsequently reduced with 1,4 Dithiothreitol (50 mM), alkylated with Iodoacetamide (50 mM), and precipitated overnight in −20 °C freezer using trichloroacetic acid (15% total volume). Protein pellets were resuspended in 40 uL of loading buffer (75% 10 mM Tris, 25% 4x loading dye [50 mM Tris HCl, 2% SDS, 10% glycerol, 0.1% bromophenol blue]) purified using SDS-PAGE (approximately 5 mm, Invitrogen NuPAGE 4–12% Bis-Tris, MOPS running buffer, 200 V constant voltage). Excised gel bands corresponding to the entire protein specimen were excised and dehydrated in successive washes with 50% acetonitrile (ACN)/50 mM ammonium bicarbonate followed by 100% ACN. Gel pieces were then subjected to in-gel tryptic digestion as previously described using sequencing grade trypsin (Promega, V5113)^20^. After digestion, each sample was desalted using Waters Sep-Pak C18 columns. Eluted peptides were then labeled with an isobaric TMT 10-plex labeling kit following the manufacturer’s recommended protocol, and desalted again. Ratios of each tag were initially verified LC/MS3 using <10% of each specimen, and then mixed accounting for deviations from the expected 1:1:1:1:1:1:1:1:1:1 ratio, with a final concentration of approximately 1 ug/ul.

### Mass spectrometry

1 uL of each sample was subject to reversed-phase chromatography on a Dionex Ultimate 3000 HPLC with an in-house laser-pulled 100 um ID nanospray column packed to ~220 mm with 3 um 2A C18 beads (Reprosil). Buffer A of the mobile phase contained 0.1% formic acid (FA) in HPLC-grade water, while buffer B contained 0.1% FA in ACN. An initial two-minute isocratic gradient flowing 3% B was followed by a linear increase up to 25% B for 115 minutes, then increased to 45% B over 15 minutes, and a final increase to 95% B over 15 minutes whereupon B was held for 6 minutes and returned back to baseline (2 min) and held for 10 minutes, for a total of 183 minutes. The HPLC flow rate was 0.400 uL/minute. The HPLC was coupled to a Thermo Fusion Lumos mass spectrometer that collected MS data in positive ion mode within the 400–1500 m/z range. A top-speed MS3 method was employed with an initial Orbitrap scan resolution of 120,000. This was followed by high-energy collision-induced dissociation and analysis in the orbitrap using “Top Speed’ dynamic identification with dynamic exclusion enabled (repeat count of 1, exclusion duration of 90 s). The automatic gain control for was set to 4e5 and 1e4 for FT full MS and ITMSn, respectively. The mass spectrometry proteomics data have been deposited to the ProteomeXchange Consortium via the PRIDE partner repository with the dataset identifier PXD012287 using ID: reviewer88334@ebi.ac.uk and password: 3woACgwT.

### Peptide/Protein search

The resulting mass spectra were searched using Proteome Discoverer 2.2. using the built-in SEQUEST search algorithm. Built-in TMT batch correction was enabled for all samples. Three FASTA databases were employed: Uniprot Swiss-Prot *Mus musculus* (taxon ID 10090, downloaded January 2017), the Human Microbiome Project database (FASTA file downloaded from https://www.hmpdacc.org/hmp/HMRGD/ on January 2017, and a database containing common preparatory contaminants. Target-decoy searching at both the peptide and protein level was employed with a strict FDR cutoff of 0.05 using the Percolator algorithm built into Proteome Discoverer 2.2^64,65^. The enzyme specificity was set to tryptic with static peptide modifications set to cambamidomethylation (+57.0214 Da) and TMT (+229.1629 Da). Dynamic modifications were set to oxidation (+15.995 Da) and n-terminal protein acetylation (+42.011 Da). Only high-confidence proteins (q-val < 0.01) were used for analysis.

### Statistical analyses

Statistics were calculated using GraphPad Prism 7, R with statistics packages (FactoMinerR 1.36, factoextra 1.0.5, ggplot2 2.2.1, Hmisc 4.0–3, psych 1.7.8, Mfuzz 2.34.0, ggpubr 0.1.5, RColorBrewer 1.1–2, UpSetR, 1.3.3, limma 3.30.13, venneuler 1.1–0), and Qlucore Omics Explorer 3.3. Data were analyzed through the use of IPA (QIAGEN Inc., https://www.qiagenbioinformatics.com/products/ingenuitypathway-analysis). Protein abundance was normalized as a percentage of summed reporter intensity for all quantified proteins in a given sample (protein intensity/total sample intensity). Missing abundance values were imputed using the lowest value found in that sample/channel. Where necessary for meeting statistical assumptions, abundances were log2 transformed (e.g. Pearson’s correlation, ANOVA). The appropriate multiple hypothesis tests were applied to abundance comparison data using Qlucore Omics Explorer or GraphPad Prism. These included one-way ANOVA with FDR correction (Qlucore), Dunn’s non-parametric test with Kruskal-Wallis correction, and two-way ANOVA with Bonferroni’s correction (Prism). Univariate analysis was conducted using Mann-Whitney U test (Prism). Correlational p-values were corrected using false discovery rate (FDR) setting and the R package psych 1.7.8. Protein abundance heat maps were generated with Qlucore Omics Explorer 3.3 with specified p-value cutoffs, FDRs and fold changes (where appropriate) were generated using Qlucore’s built-in FDR estimator, and the values are reported in Supplementary Tables.

## Supplementary information

Supplementary Info

Supplementary table 2

Supplementary table 3

Supplementary table 4

Supplementary table 5

Supplementary table 6

Supplementary table 7

Supplementary table 8
