# Supplementary material for: Latent-period stool proteomic assay of multiple sclerosis model indicates protective capacity of host-expressed protease inhibitors
Source: Sci Rep. 2019 Aug 28;9:12460. doi: 10.1038/s41598-019-48495-5 (PMC6713765; doi:10.1038/s41598-019-48495-5)
Supplement: Supplementary file 1 — Supplementary Info [file 41598_2019_48495_MOESM1_ESM.pdf]

Latent-period stool proteomic assay of multiple sclerosis model indicates protective capacity of host-expressed protease inhibitors.

Carlos G. Gonzalez[1], Stephanie K. Tankou[2-5], Laura M. Cox[2], Ellen P. Casavant[1], Howard L. Weiner[2], Joshua E. Elias*[1].

[1]Chemical and Systems Biology Department, Stanford University School of Medicine, [2]Ann Romney Center for Neurological Disorders, Brigham and Women's Hospital, Harvard School of Medicine. [3]Department Of Neurology, Icahn School Of medicine at Mount Sinai, New York, NY, USA [4]Precision Immunology Institute, Icahn School Of medicine at Mount Sinai, New York, NY, USA [5]Friedman Brain Institute, Icahn School Of medicine at Mount Sinai, New York, NY, USA *Correspondence to jee3@stanford.edu.

Supplemental Figures/Tables:

Animal	M1	M2	M3	M4	M5	M6	M7	M8
EAE max score	4	3	0.5	1.5	0	1.5	3	3
Day of disease onset	10	10	N/A	13	N/A	13	11	10
EAE cumulative score	13.5	8.5	0.5	1.5	0	2	8	8.5

SI. Table 1: EAE scoring and metrics. EAE metrics including maximum score observed, cumulative score, and disease of onset for each individual mouse. Note that since VT mice and CFA/PTX-only mice did not develop EAE, no score is recorded.

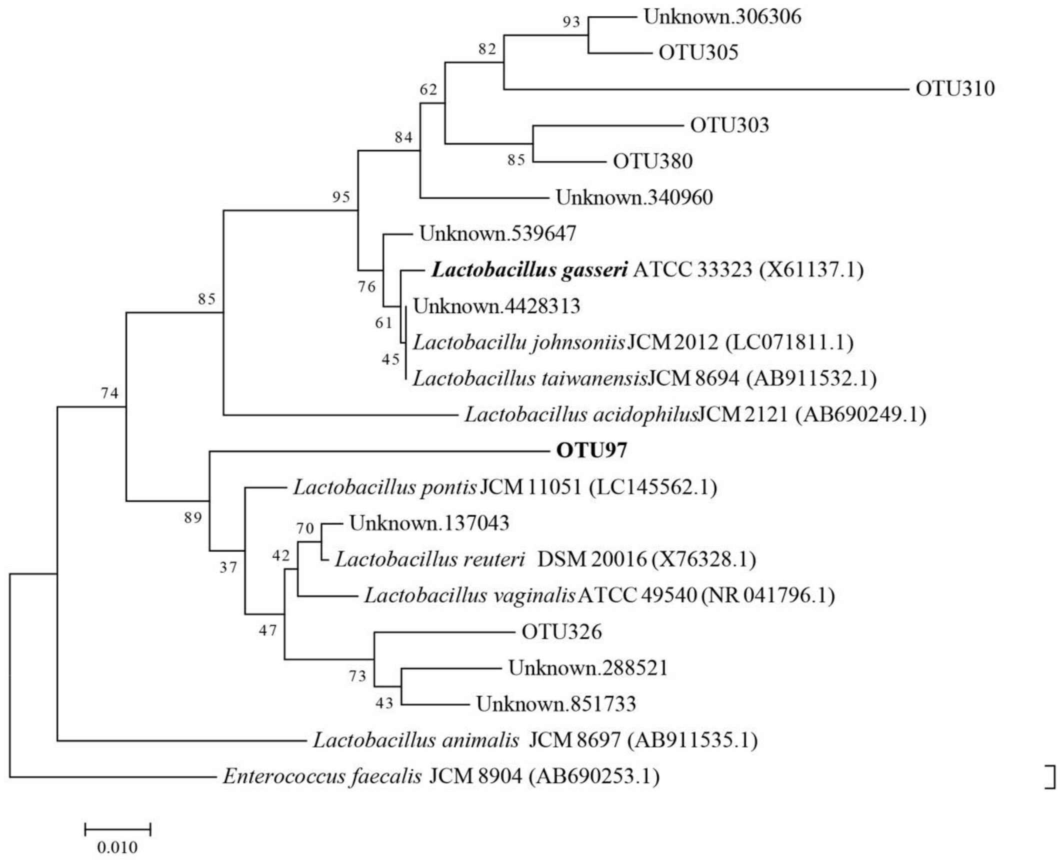

SI. 1: Phylogenetic distance map of Lactobacillus species constructed from 16S rRNA sequencing and targeted analysis. The limited taxonomic resolution of Lactobacillus beyond the genus level was supplemented with orthogonal taxon-picking algorithms, including BLAST and MEGA7 to elucidate unresolved taxa [17,18].

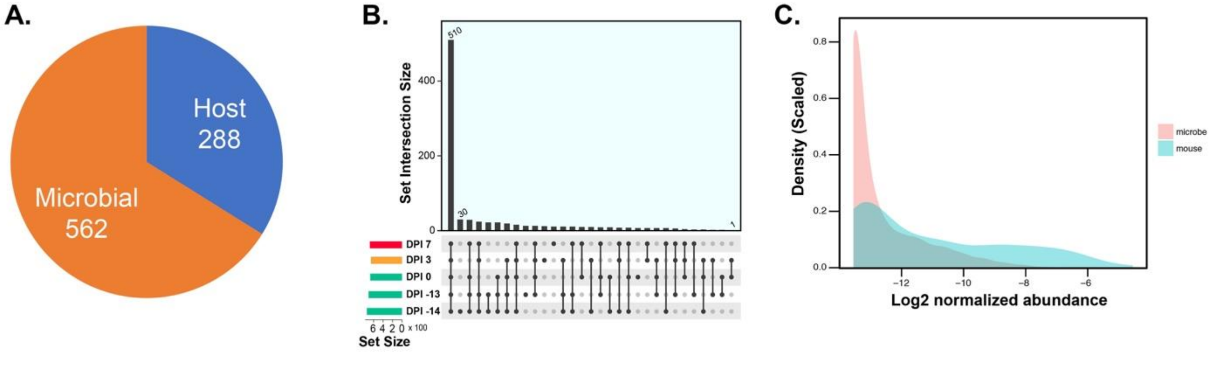

SI. 2: Protein metrics suggest shotgun metaproteomics results in reliable measurements of latent period EAE extracellular stool proteome. A. Breakdown of

proteins attributed to host or microbes in our analysis. **B.** Combinatorial set analysis of all detected host and microbe proteins. Blue horizontal bars indicate overall day set size, while black vertical bars represent set size present in specific subset highlighted by connected black dots below bar. **C**. Log2 normalized protein abundance density plot indicates host proteins are far more abundant than microbial proteins in the extracellular stool proteome.

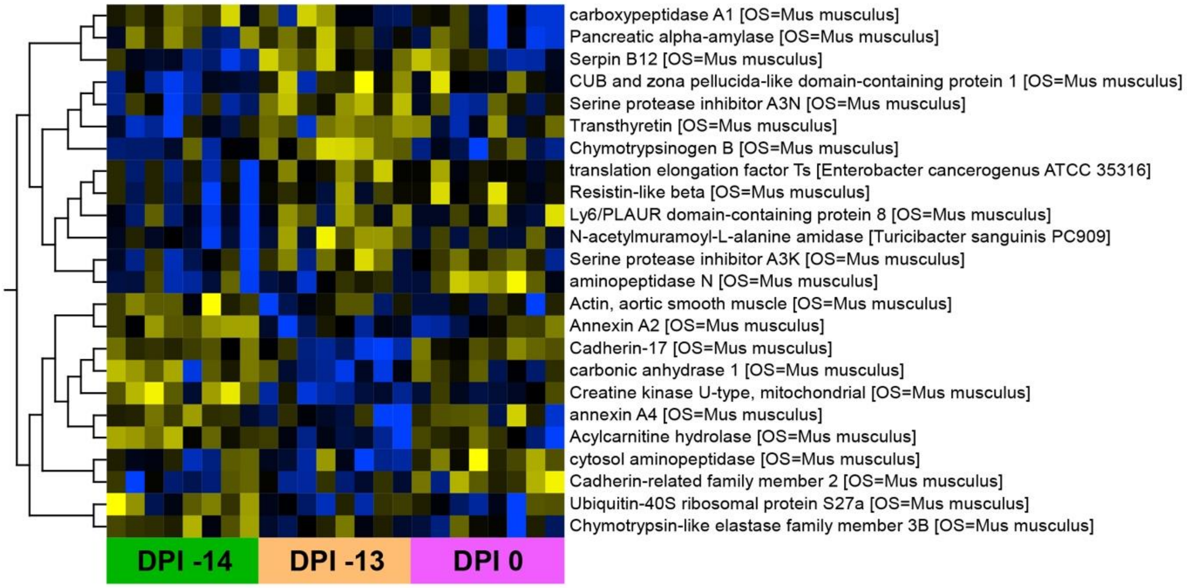

SI 3. Heatmap of pre-immunized stool proteome. Heatmap generated using significantly altered ($p < 0.05$, ANOVA) normalized and log2 transformed protein abundance. Heatmap sorted in chronological order (n = 8 mice per timepoint, x-axis) and hierarchical clustering of protein abundances (y-axis).

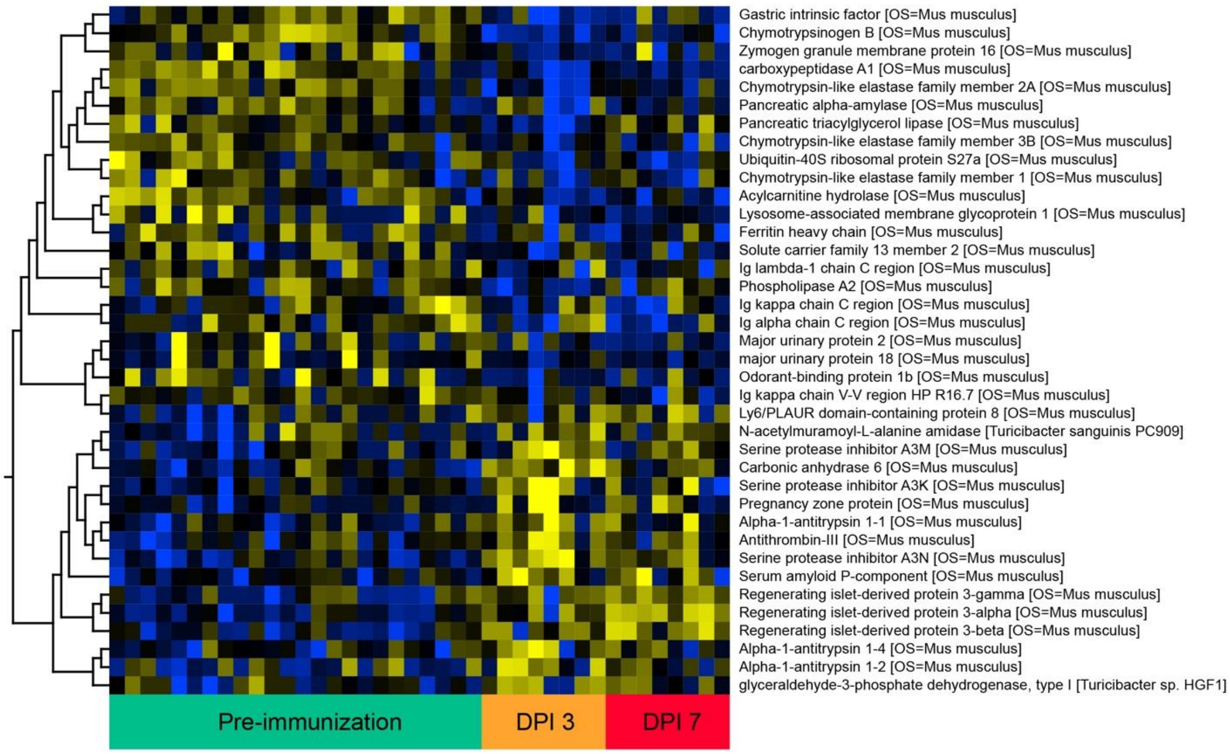

SI. 4. Heatmap of post-immunized stool proteome. Heatmap generated using significantly altered ($p < 0.05$, ANOVA) normalized and log2 transformed protein abundance. Heatmap sorted in chronological order (n = 8 mice per timepoint, x-axis) and hierarchical clustering of protein abundances (y-axis); full statistics available in table 1.

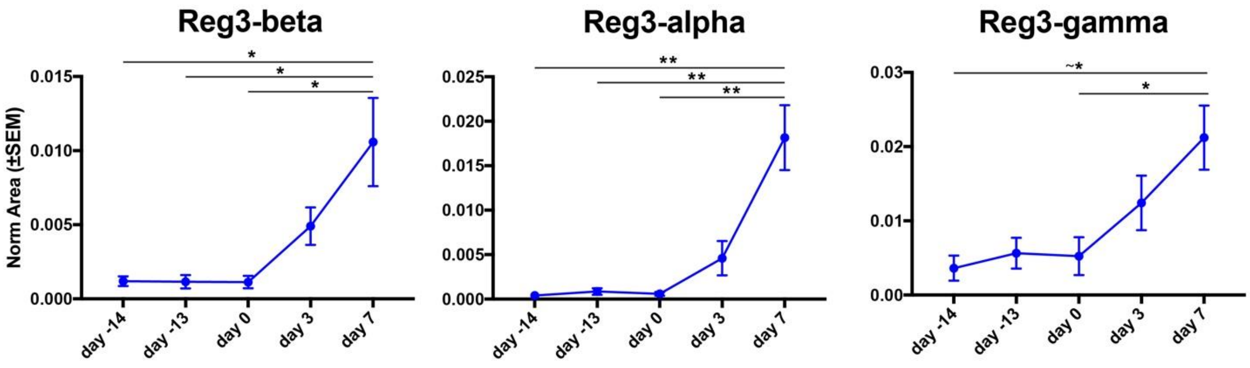

SI. 5: Univariate analysis of Reg3-family lechtins reveals increase after immunization leading up to symptomatic phase. One-way ANOVA (Dunn's significance, ~* $p = 0.058$, * $p < 0.05$, ** $p < 0.01$) of Reg3-ß (Kruskal-Wallis statistic = 1

7.21), Reg3-α (Kruskal-Wallis statistic = 22.2), and Reg3-γ (Kruskal-Wallis statistic = 12.7) abundance over the course of the study.

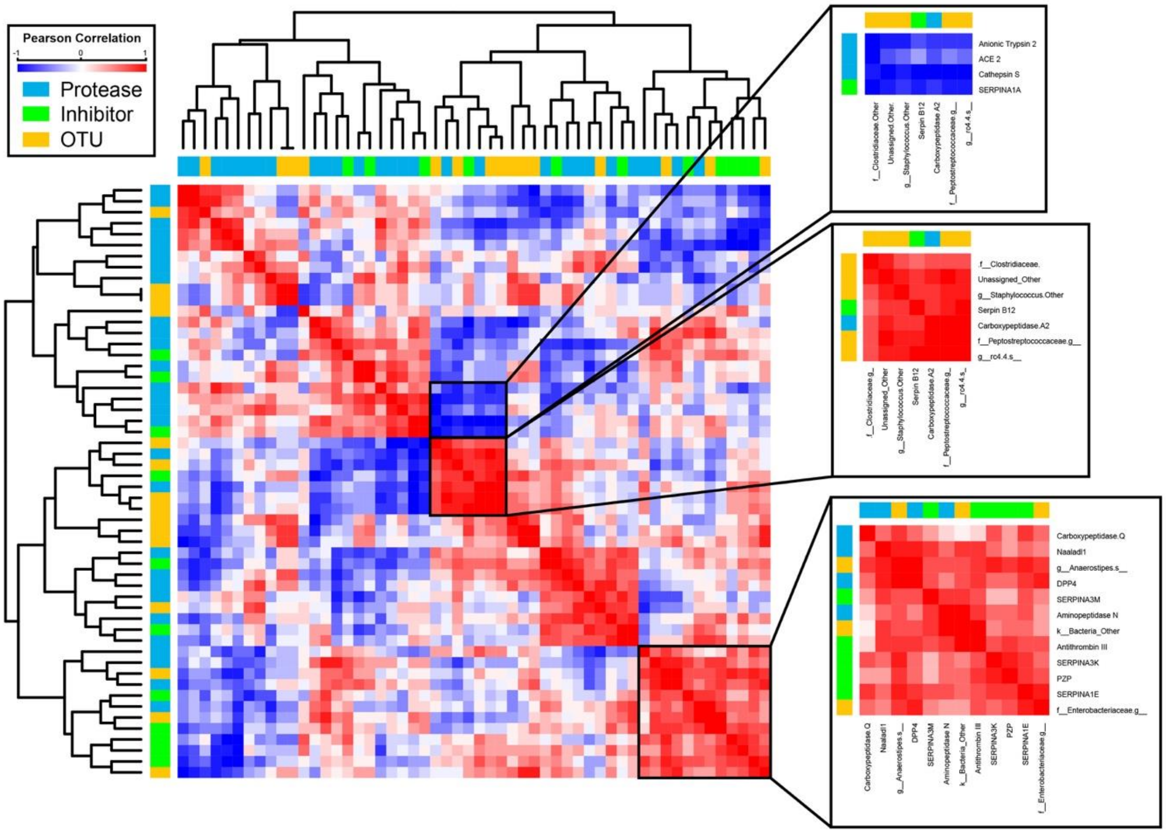

SI. 6. Correlation analysis of proteases, protease inhibitors, and OTUs on DPI 3. All maps generated using log2-transformed data (n=6 due to missing 16s rRNA data) and correlated using Pearson correlations. OTUs were selected by a p-value cutoff (< 0.05) and fold-change threshold of 1.5x fold change post-immunization Hierarchical clustering used to sort protein-OTU pairs by abundance similarities.

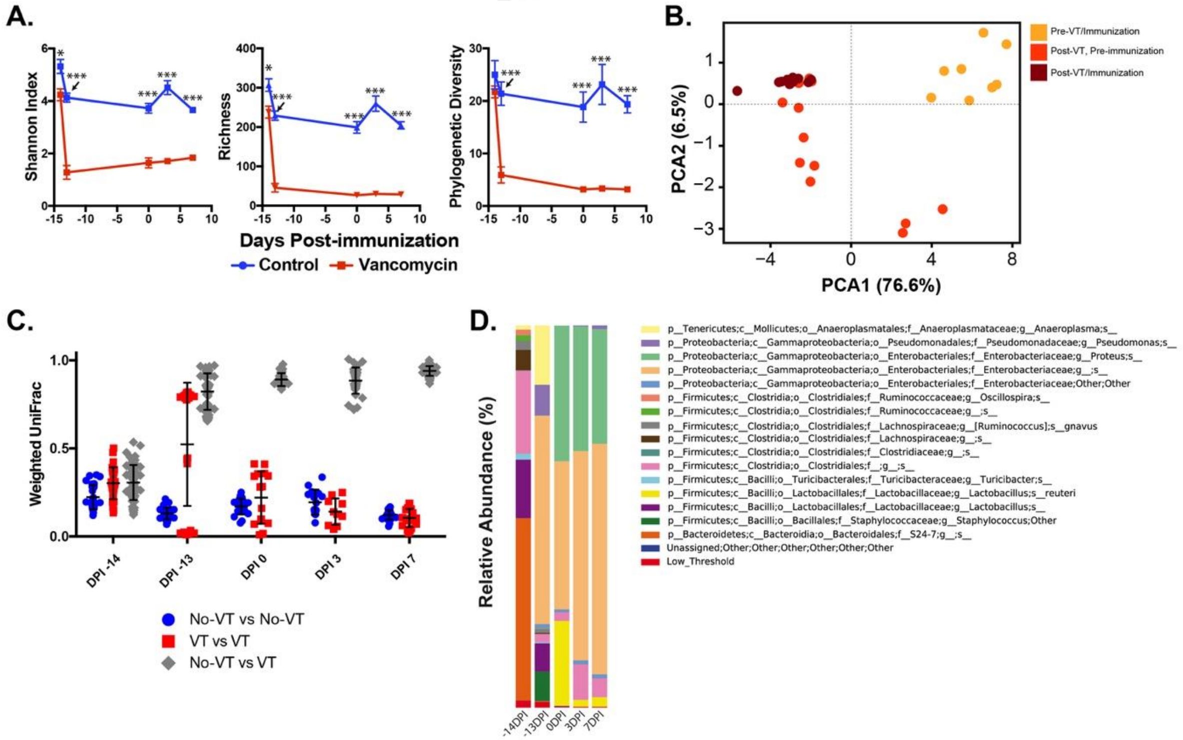

SI. 7. **Vancomycin administration alters microbiome community over the course of study**. **A.** Indices of community structure comparing untreated group previously described (n=8) and VT group (n=8) over time (Bonferroni corrected p-values = *$p < 0.05$, **$p < 0.01$, ***$p < 0.001$; F-values available on Supplementary table 8). **B.** PCA generated using microbes significantly altered between days (ANOVA, $p < 0.05$; F(10,57 = 2.007)). **C.** One-way ANOVA with Dunnett's post-test comparing each group against baseline Weighted UniFrac distances. **D.** Mean relative abundance (%) of top 18 OTU in VT mice over the course of the study.

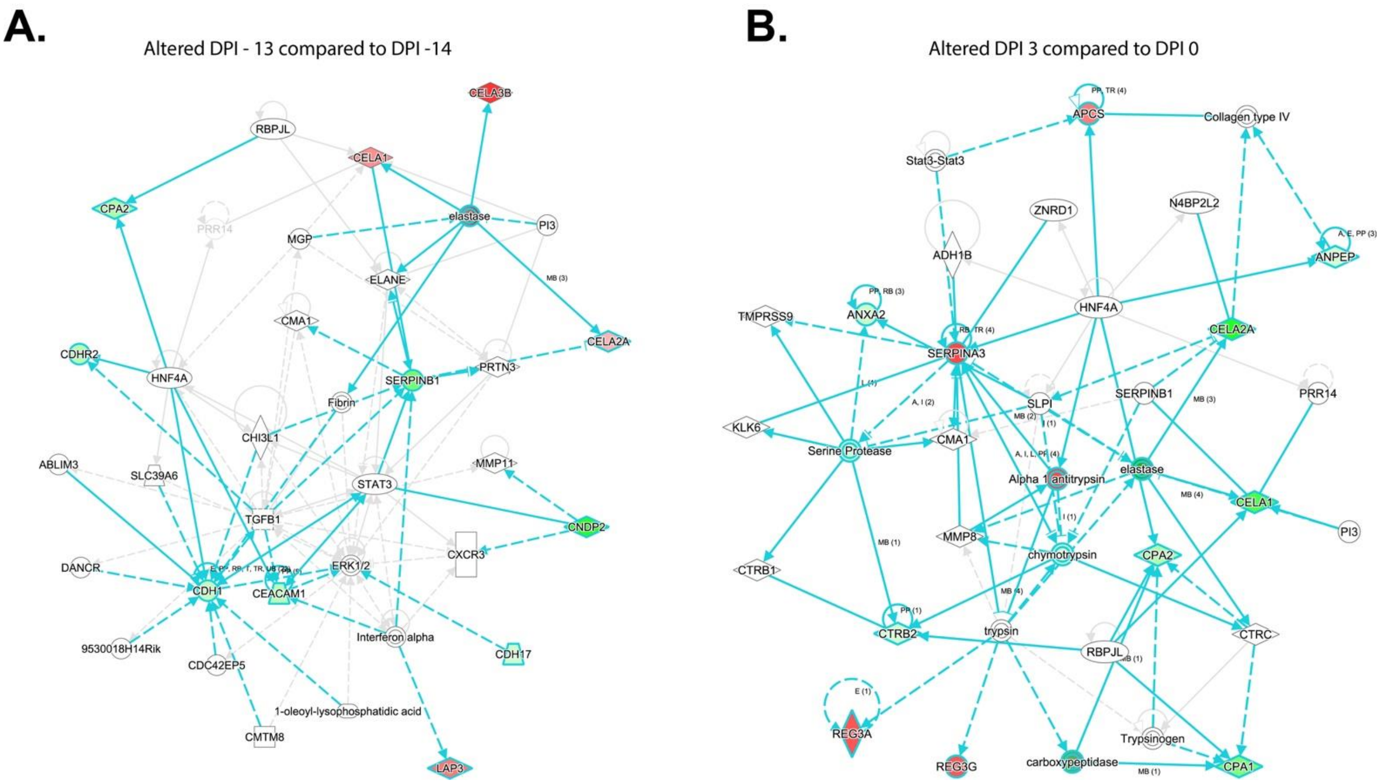

SI. 8. IPA network analysis of proteins altered on DPI -13 (A) and DPI 3 (B). Using a statistical cutoff ($p < 0.05$), protein abundance was used to generate a protein interaction network. Solid lines represent direct relations while dotted lines represent indirect relationships. Proteins highlighted in green represent proteins decreased on that day compared to the previous time point, while proteins highlighted in red represent increased in proteins on that day compared to the previous time point. The networks were generated through the use of IPA (QIAGEN Inc., https://www.qiagenbioinformatics.com/products/ingenuity-pathway-analysis).

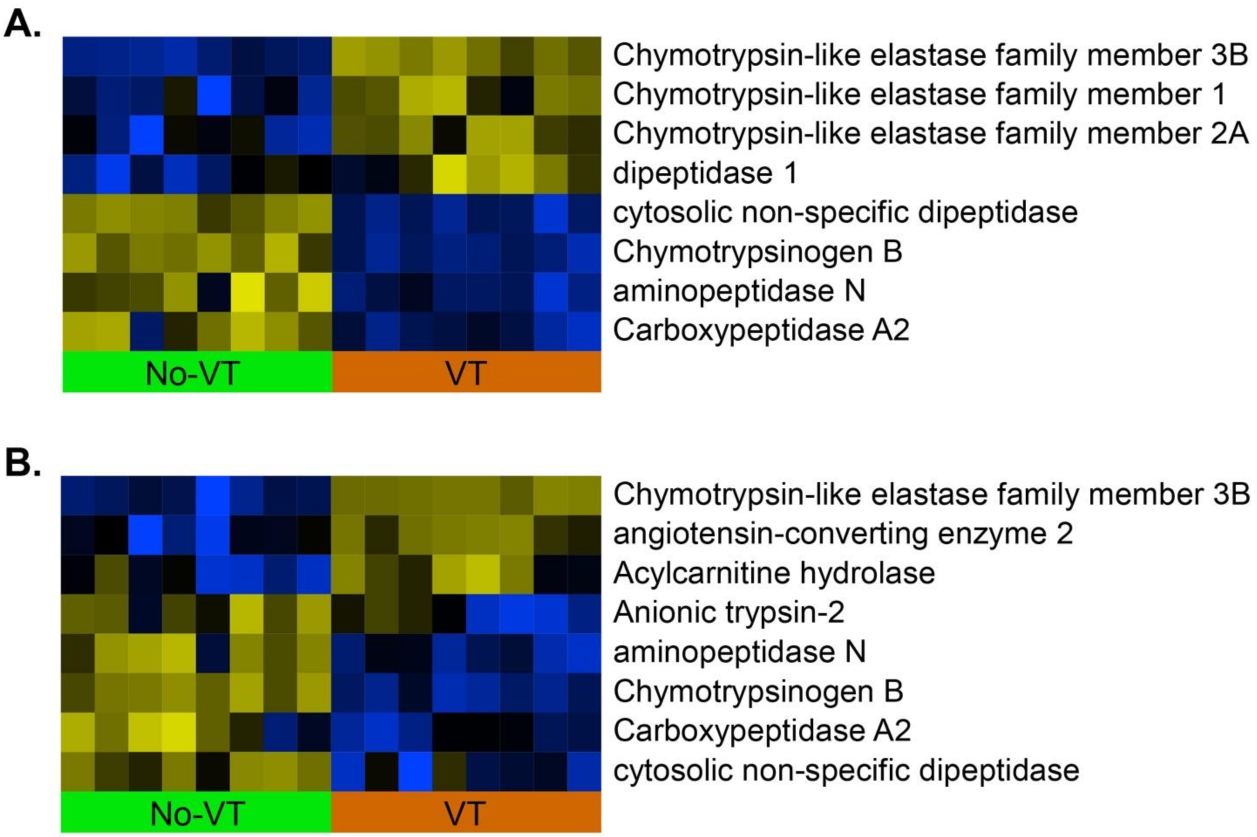

SI. 9. Comparison of differentially expressed proteases between VT and no-VT mice on either DPI -13 (A) or DPI 3 (B). Proteases were selected using a one-way ANOVA with a significance cutoff of $p < 0.05$.

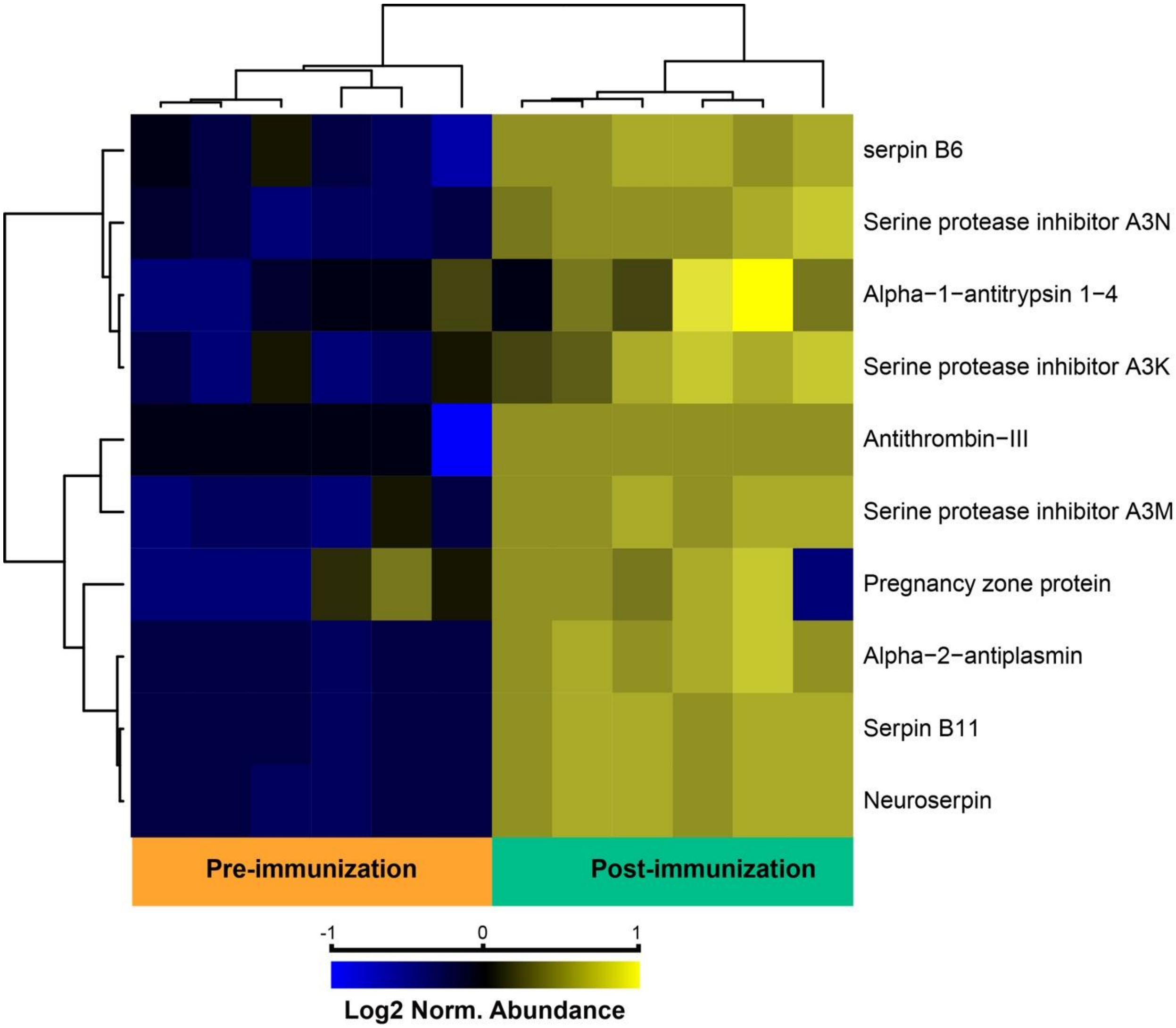

SI. 10. CFA/CT adjuvant alone recapitulates increase in protease inhibitors post-immunization. Stool from DPI 0 was compared to DPI 3 in six mice receiving CTA/CT adjuvant without MOG. Log2-normalized protease inhibitors that were significantly (t-test, $p < 0.05$; for complete statistics see Supplementary Table 8).

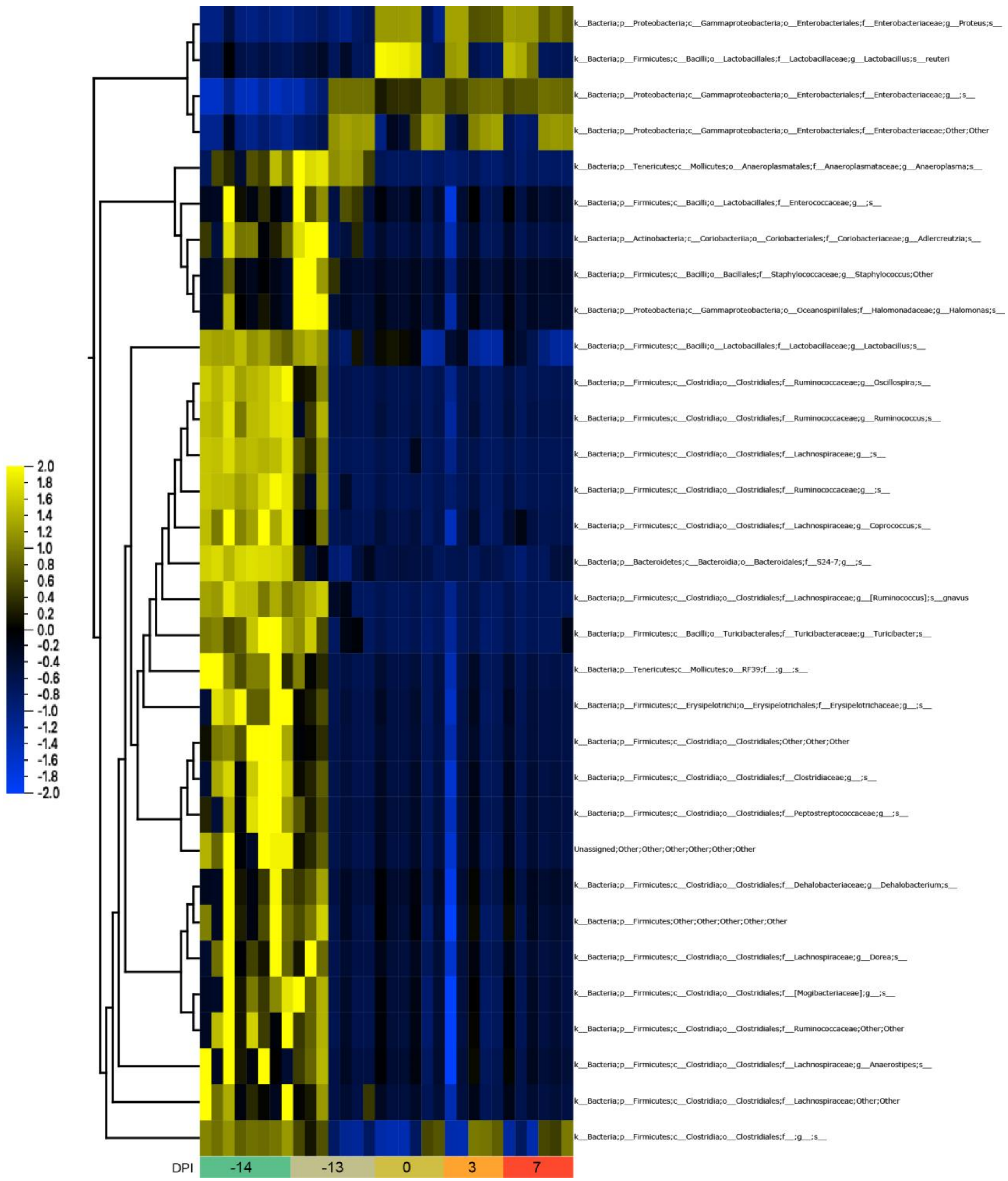

SI. 11. Vancomycin modulates microbial abundance. Heatmap of significantly altered microbe (32/91 microbes altered) abundances on DPI -14 (n=8) compared to DPI -13 (n=7), 0 (n=, 5), 3 (n=6), or 7 (n=6) ($p < 0.05$, Log2 transformed, ANOVA). Vancomycin was administered beginning DPI -14 after stool collection. Each column

represents a separate mouse. Mice are grouped by color-coded days post immunization (DPI) in chronological order.

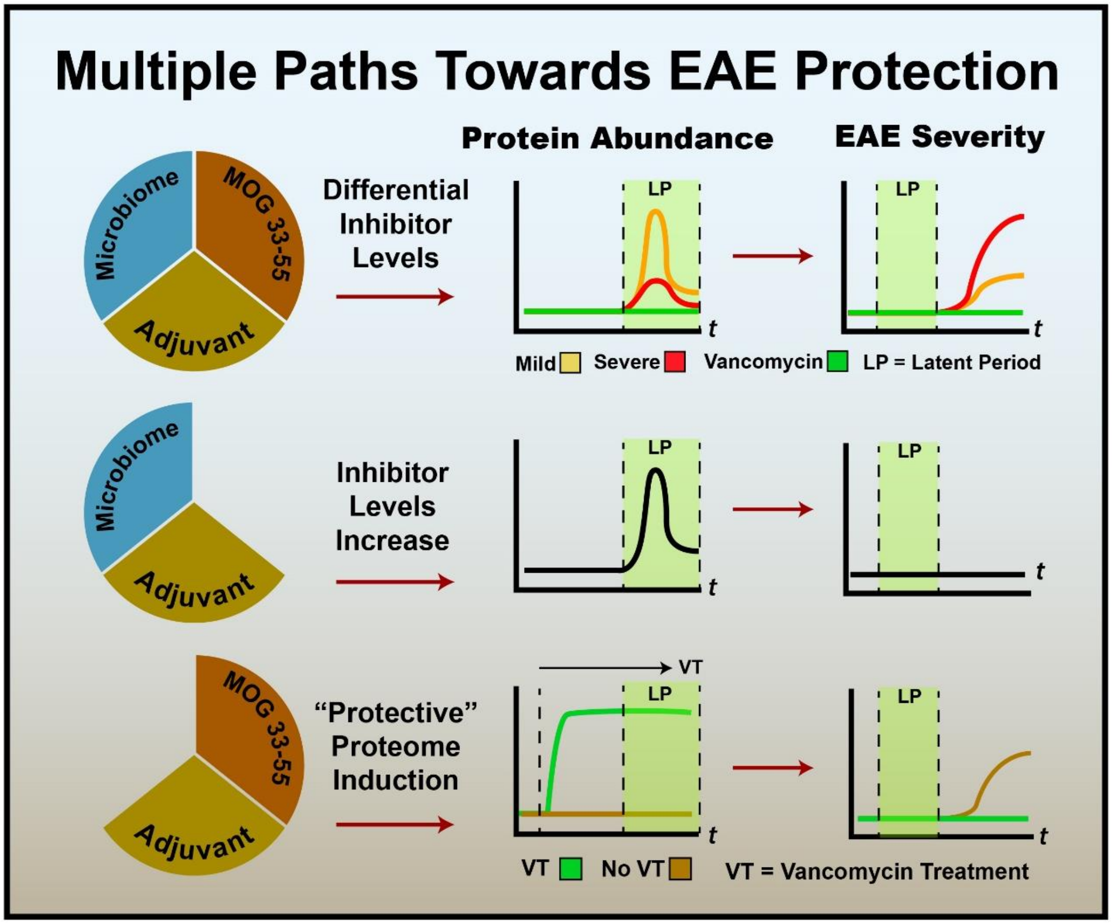

SI. 12. Summary model of findings for both VT and non VT-mice. Our multi-omic findings suggest there are multiple pathways that can affect EAE outcome, and these are assayable using the stool proteome.
